# Neuropilin 1 Is Essential for Gastrointestinal Smooth Muscle Contractility and Motility in Aged Mice

**DOI:** 10.1371/journal.pone.0115563

**Published:** 2015-02-06

**Authors:** Maiko Yamaji, Marwa Mahmoud, Ian M. Evans, Ian C. Zachary

**Affiliations:** Centre for Cardiovascular Biology and Medicine, Division of Medicine, the Rayne Building, University College London, London, WC1E 6JJ, United Kingdom; Temple University School of Medicine, UNITED STATES

## Abstract

**Background and Aims:**

Neuropilin 1 (NRP1) is a non-tyrosine kinase receptor for vascular endothelial growth factor (VEGF) and class 3 semaphorins, playing a role in angiogenesis and neuronal axon guidance, respectively. NRP1 is expressed in smooth muscle cells (SMC) but the functional role of NRP1 in SMC has not been elucidated. We therefore investigated the biological relevance of NRP1 in SMC *in vivo* by generating mice with SMC-specific *Nrp1* deficiency.

**Methods:**

Conditional gene targeting generated SMC-specific *Nrp1* knockout mice (*Nrp1^SMKO^*) in which Cre recombinase is driven by the smooth muscle-specific myosin heavy chain (*smMHC*) promoter.

**Results:**

SMC-specific *Nrp1* deficiency resulted in a significant reduction in intestinal length by 6 months, and, by 18 months, in severe constipation, and enlargement of the intestine consistent with chronic intestinal pseudo-obstruction. These effects were associated with significant thinning of the intestinal smooth muscle, and decreased intestinal contractility. Expression of contractile proteins was reduced in *Nrp1^SMKO^* mice, including the smMHC isoform, SMB, whereas we observed a significant increase in the expression of the small-conductance calcium-activated potassium channel 3 (SK3/KCa2.3), implicated in negative regulation of smooth muscle contraction.

**Conclusions:**

*Nrp1* deficiency in visceral SMC results in adult-onset defects in gastrointestinal contractility and motility and causes a shift to a less contractile SMC phenotype. These findings indicate a new role for *Nrp1* in the maintenance of the visceral SMC contractile phenotype required for normal GI motility in aged mice.

## Introduction

Neuropilin 1 (NRP1) is a transmembrane glycoprotein receptor for two types of ligands, the class 3 semaphorins which regulate axonal guidance and neuronal patterning during development [1,2], and members of the VEGF family of angiogenic cytokines in endothelial cells [3]. *Nrp1*-null mice die between embryonic day (E) 10.5 and E14.5, dependent on the genetic background, with a spectrum of cardiovascular and neuronal defects [4,5]. Mice with loss of *Nrp1* in vascular endothelial cells are also embryonic lethal due to aberrant cardiovascular development [6]. Mice heterozygous for *Nrp1* are viable and fertile with no discernible spontaneous phenotype, but exhibit increased mortality when challenged by cardiac pressure overload [7]. The precise cellular function of NRP1 is unclear, but it plays an essential role in endothelial cell motility [8,9,10], and in growth cone collapse in sensory neurons [1].

Recent evidence indicates that NRP1 is expressed in other cell types [8], including smooth muscle cells (SMC) [11,12,13,14,15]. NRP1 is highly expressed in human aortic and coronary artery SMC [12,13,14] and inhibition of NRP1 function decreases the migratory and signaling responses of SMC to PDGF-BB [12]. *In vivo*, apart from neurons, epicardium and endothelium, Nrp1 is reported to be most strongly expressed in late mouse embryos in the SMC of large vessels [15]. However, the functional role of NRP1 in SMC either in the vasculature, the gastrointestinal (GI) tract or in other organs is currently unknown.

Smooth muscle is essential for the contractility of blood vessels, the uterus, and the organs of the GI tract, including the small and large intestines, the bladder and the urinary tract. Peristaltic gut motility, essential for the movement and digestion of food, and the excretion of solid waste, occurs as a result of the co-ordinated action of the circular and longitudinal smooth muscle layers in the GI wall. Genetically-targeted deficiency of components of the smooth muscle contractile apparatus, such as myosin light chain kinase (MLCK) [16], or smoothelin [17], causes defects in gut smooth muscle organisation associated with impaired GI contractility and motility.

Since NRP1 is expressed in SMC, we sought to investigate its role in SMC functions in vivo by generating mice with a SMC-specific loss of Nrp1 (Nrp1SMKO). These mice were viable, healthy, with no overt phenotype during early post-natal life. Unexpectedly, however, Nrp1SMKO mice developed a pronounced age-dependent defect in GI motility. Further studies of GI SMC function in aged Nrp1SMKO mice revealed marked defects in intestinal length and SMC architecture, combined with impaired GI motility and SMC contractility. These defects in GI tissue and function were associated with changes in expression of markers of SMC phenotypes indicating that loss of Nrp1 caused a switch to a less contractile SMC phenotype. Furthermore, examination of early post-natal mice revealed changes indicative of a similar early shift to a less contractile phenotype in Nrp1SMKO mice. These results show that NRP1 is essential for maintenance of the visceral SMC contractile phenotype and of the contractile functions of SMC required for normal GI motility

## Materials and Methods

This work was performed under UK Home Office Licence 70/7700, following full ethical approval by the University College London (UCL) Animal Welfare and Ethical Review Body, and review by the UK Home Office.

### Generation of SMC specific *Nrp1* knockout mice

**Figure 1:**
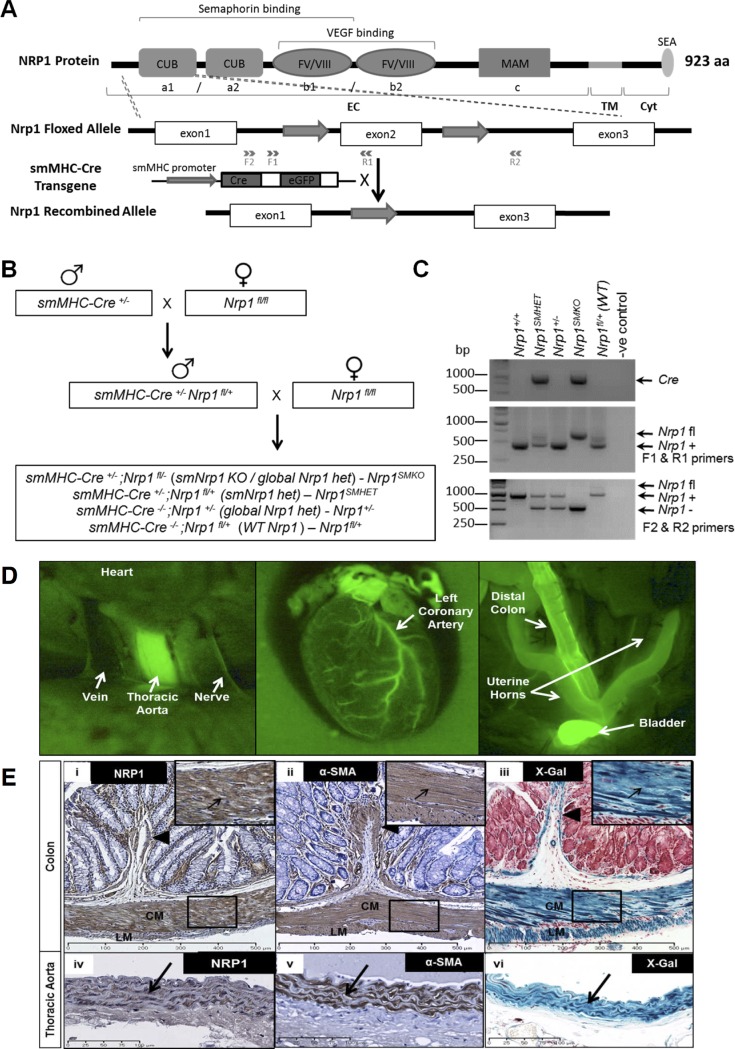
Conditional targeting of *Nrp1* in SMC. (A) Schematic diagram of the targeting strategy to generate *Nrp1^SMKO^* mice. The structure of the NRP1 protein shows the major domains in the extracellular (EC), the transmembrane (TM) and cytoplasmic (Cyt) domains. In floxed *Nrp1* (*Nrp1* ^fl^) mice, the exon2 of *Nrp1* is flanked by two loxP sites, which undergo Cre-mediated recombination in SMC when crossed to the *smMHC-Cre* mice, resulting in a null *Nrp1* allele. The positions of primers (F1,F2,R1,R2) used for genotyping are indicated. (B) Breeding scheme used to generate *Nrp1^SMKO^* and heterozygous *Nrp1^+/−^* controls. In the second round of breeding four different genotypes are generated as indicated in parentheses: *Nrp1^fl/+^* (WT), *Nrp1^SMHET^*, *Nrp1^+/−^*, and *Nrp1^SMKO^*. (C) Genotyping of ear notch DNA. A 900bp band indicates the presence of the *Cre* transgene (top gel). PCR with primers F1 and R1 (middle gel) detects the loxP site and generates 486bp (*Nrp1* WT) and 700bp (*Nrp1* ^fl^, floxed) products. PCR with primers F2 and R2 (bottom gel) detects Cre-mediated recombination of floxed *Nrp1* and generates 986bp (*Nrp1* WT), ∼600bp (*Nrp1* ^−^, recombined) and ∼1200bp (*Nrp1* ^fl^, unrecombined). (D) GFP fluorescence in *smMHC-Cre* ^+/−^ (green) shows that Cre recombinase expression is restricted to vascular and visceral SMC. (E) NRP1/SMA immunohistochemistry and X-gal staining of adult colon and thoracic aorta. NRP1 (i,iv) and SMA (ii, v), are expressed in both the circular (CM) and longitudinal (LM) muscle layers, and the muscularis mucosae (arrowhead) of the colon and in the tunica media of the thoracic aorta (arrow). (iii, vi) X-gal staining of sections from a *smMHC-Cre/Rosa26R* double heterozygote shows that the expression of the lacZ (blue), driven by the smMHC promoter, is restricted to visceral (iii) and vascular (vi) SMC. Boxed regions are magnified views showing expression of NRP1, SMA and LacZ in smooth muscle cells of the colon.

Animal experiments were conducted in accordance with the Animal Care and Ethics Guidelines of University College London (UCL) and the United Kingdom Home Office Animals (Scientific Procedures) Act (1986). In order to generate SMC-specific *Nrp1* knockout mice, two successive rounds of breeding were performed (Fig. 1*B*). First, we crossed heterozygous male smooth muscle myosin heavy chain (smMHC)-Cre/enhanced green fluorescent protein (eGFP) transgenic mice (*smMHC-Cre^+/−^*) [18,19,20] with homozygous female floxed *Nrp1 (Nrp1^fl/fl^)* mice [6]. The resulting offspring that are SMC-specific *Nrp1* heterozygotes (*smMHC-Cre^+/−^; Nrp1^fl/+^*) were healthy, viable and fertile. Male *smMHC-Cre^+/−^; Nrp1^fl/+^* mice, that were generated from the first cross, were then bred with female *Nrp1^fl/fl^* mice to generate SMC-specific *Nrp1* knockout mice (*smMHC-Cre^+/−^; Nrp1^fl/fl^*). In smMHC-Cre/eGFP transgenic mice, transient expression of Cre recombinase occurs in the germ cells with expression ending prior to fertilization [19]. Therefore, any floxed allele transmitted from the Cre-expressing parent will be recombined (whether the haploid male germ cell is Cre-positive or Cre-negative), resulting in a null allele of *Nrp1* (hereafter referred to as -) in all tissues and giving rise to mice heterozygous for global *Nrp1* deficiency. Since Cre activity stops prior to fertilization, the resulting offspring will exhibit no universal deletion of any floxed allele of maternal origin. Consequently, in the second breeding round four different genotypes were generated: *smMHC-Cre^+/−^;Nrp1^fl/−^* (SMC-specific *Nrp1* knockout and also globally heterozygous for *Nrp1*; hereafter designated *Nrp1^SMKO^*), *smMHC-Cre^−/−^;Nrp1^fl/−^* (globally *Nrp1* heterozygous, designated *Nrp1^+/−^*) which we used as the controls for the SMC-specific *Nrp1* knockout and globally *Nrp1* heterozygous mice (*Nrp1^SMKO^*); *smMHC-Cre^+/−^;Nrp1^fl/+^* (SMC-specific *Nrp1* heterozygous, designated *Nrp1^SMHET^*), and *smMHC-Cre^−/−^;Nrp1^fl/+^* (designated *Nrp1^fl/+^* or WT). Genotyping of genomic DNA from ear biopsies was performed by polymerase chain reaction (PCR) (S1 Table).

### Protein and Gene Expression

Protein expression was determined in paraffin-embedded sections by immunostaining staining, and in tissue lysates by immunoblotting, as described [21,22], using primary and conjugated secondary antibodies as indicated in S2 and S3 Tables. Levels of mRNA were measured in tissue RNA by quantitative real-time PCR (qPCR) using the primers indicated (S4 Table) as described [22,23], or by semi-quantitative reverse-transcription PCR (RT-PCR).

### *In vivo* BrdU labelling

Proliferating cells in the colon were detected in P4, P7 and adult (6M) mice by immunostaining bromodeoxyuridine (BrdU) following intraperitoneal BrdU injection. To detect proliferating cells, each mouse was injected intraperitoneally with 100μg/g body weight bromodeoxyuridine (BrdU) (Sigma, #B5002) 4 hours before harvesting the tissue. Tissue sections were treated with 2M HCl for 45 minutes followed by incubation with 0.1M Boric acid for 30 minutes prior to immunohistochemistry with anti-BrdU antibody (Dako # M0744). Proliferating cells were counted in the muscularis externa of the colon,

### Measurement of gastric emptying by 24 hour stool collection

Assessment of gastric emptying was carried out as described before [24, 25], except that the monitoring period was extended to 24 hours. Briefly, mice were placed in a separate clean cage with regular diet (Harlan) and the faecal output was screened by counting all the faecal pellets collected after a 24 hour-period. Six faecal pellets from an individual mouse were placed in an eppendorf tube, air-dried overnight and weighed and measured by an electronic digital caliper.

### Measurements of intestinal contractility

Colonic contractility was measured in organ baths containing Krebs-Ringer buffer at 37°C. Colonic segments were stretched gradually to .5–1.0g tension and challenged with 80mM potassium chloride (KCl). Increasing doses of carbachol (CCh) or KCl were then administered. Changes in isometric tension were measured and recorded by LabChart software (AD Instruments Ltd., Oxford).

### Statistical analyses

Statistical significance was assessed either by repeated measures two-way analysis of variance followed by Bonferroni’s Multiple Comparison Test, or two-tailed Student *t* test. When the interaction was found significant in repeated measures two-way analysis of variance, one-tailed paired Student *t* test was performed. The significance of association between SMB isoform expression and the genotype was analysed in a 2×2 contingency table using a chi-squared test. *P<*.05 was considered statistically significant.

## Results

### SMC-specific targeting of *Nrp1* in mice

The smMHC-Cre/eGFP mouse line, expressing Cre recombinase under the control of the smooth muscle-specific myosin heavy chain promoter [18,20], has been widely used to create SMC-specific conditional gene deletions [26,27]. Therefore, to determine the role of Nrp1 in SMC *in vivo* we generated mice harbouring a deletion of *Nrp1* in SMC by crossing *Nrp1* floxed mice [6] with smMHC-Cre/eGFP transgenic mice (Fig. 1*A-C*). Analysis of over 100 offspring from 15 breeding groups showed that the genotypes of weaned animals occurred close to expected Mendelian ratios (S5 Table).

Determination of eGFP expression in smMHC-Cre/eGFP mice confirmed that Cre recombinase activity was restricted to smooth muscle-rich tissues, including the large arteries, colon, bladder and uterus (Fig. 1*D*). To further verify restriction of Cre recombinase activity to SMC, smMHC-Cre/eGFP mice were crossed with ROSA26 Cre reporter mice [28]. Analysis of beta-galactosidase activity in the progeny of this cross demonstrated efficient recombination of the ROSA26 reporter allele in SMC-rich regions of the colon and thoracic aorta; additionally, immunohostochemical staining of SMC-specific alpha-actin (SMA) and NRP1 on serial tissue sections confirmed the co-localisation of NRP1-positive SMC with beta-galactosidase activity in these tissues (Fig. 1*E*).

**Figure 2:**
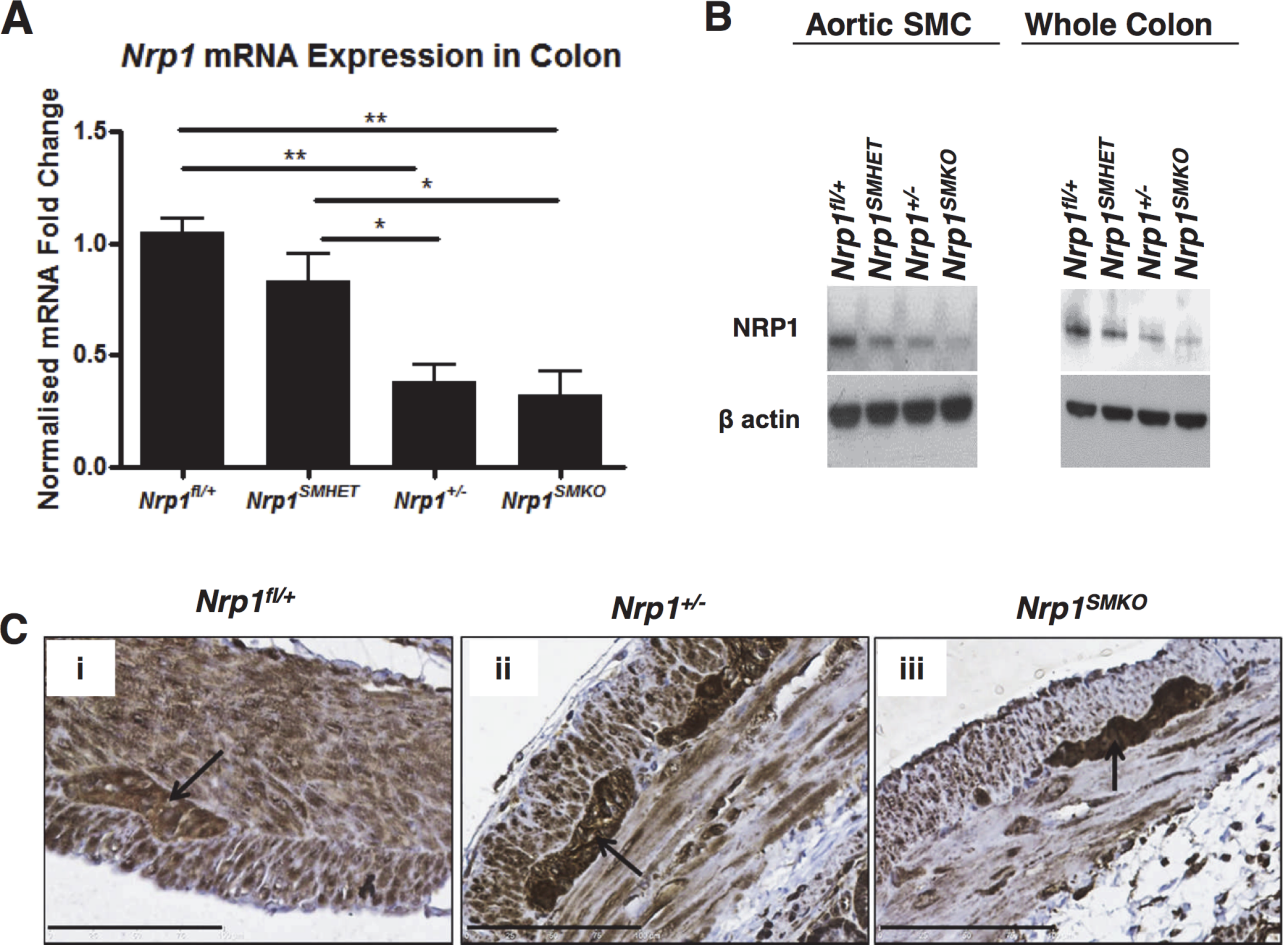
Reduced SMC-specific *Nrp1* expression in *Nrp1^SMKO^*. (A) qPCR analysis on the whole adult colon: means±SD, n = 3, *P<.05, **P<.005, ***P<.001. (B) Western blots of NRP1 in the isolated smooth muscle layer from the thoracic aorta and in whole colon tissue samples from mice >18 months old. (C) Reduced expression of NRP1 in visceral SMCs is detected by immunohistochemical staining of adult colon tissue sections. Reduced expression, specifically in SMCs, is evident in the *Nrp1^SMKO^* (iii) sample when compared to the *Nrp1^fl/+^* (i) and *Nrp1^+/−^* (ii) samples, and no change in NRP1 expression can be seen in the myenteric plexus of Auerbach (indicated by the arrows).

The efficiency of recombination of the *Nrp1* floxed alleles by Cre recombinase was verified by measurement of *Nrp1* mRNA and protein expression. *Nrp1* mRNA levels in the colon, measured using qPCR, were markedly reduced both in mice that had a SMC-specific *Nrp1* knockout and were also globally heterozygous for *Nrp1* (*Nrp1^SMKO^*) (32.87% ± 10.7%) and in globally heterozygous Nrp1 mice (*Nrp1^+/−^*) (38.93% ± 4.99%), as compared to wild-type *Nrp1^fl/+^* (100%) (identical to *Nrp1^+/fl^* genotype; see Fig.1B) mice (Fig. 2*A*). The similar loss of *Nrp1* mRNA expression in *Nrp1^SMKO^* and globally heterozygous *Nrp1^+/−^* mice probably reflects significant expression of *Nrp1* in non-SMC cells in the colon such as vascular endothelium and sensory neurons. Western blotting revealed a striking reduction in NRP1 protein expression in aortic SMC isolated from thoracic aortae and in whole colon protein lysates in *Nrp1^SMKO^* as compared to *Nrp1^+/+^* mice, which was greater than the loss of NRP1 in *Nrp1^+/−^* global heterozygotes (Fig. 2*B*). Quantification of protein expression in western blots showed that in *Nrp1^SMKO^* mice as compared to *Nrp1^+/−^* heterozygotes, colonic NRP1 expression was significantly reduced in adult (>18 months) mice (S1A Fig.). Colonic NRP1 protein expression was also significantly reduced in *Nrp1^+/−^* heterozygotes (*p* < 0.001) and in *Nrp1^SMKO^* (*p* < 0.001) post-natal day (P) 7 pups as compared to wild-type (WT) *Nrp1^fl/+^* pups. Immunohistochemical staining for NRP1 also revealed a marked reduction in NRP1 expression in the visceral SMC of colons from *Nrp1^SMKO^* compared with WT *Nrp1^fl/+^* mice and *Nrp1^+/−^* heterozygotes (Fig. 2C). Double immunofluorescent staining in colon sections for NRP1 and SMA also showed co-staining of NRP1 expression and SMA in smooth muscle layers of the colon in WT mice (S1B Fig.). We also cultured visceral SMCs from smooth muscle strips prepared from the bladders of P7 neonatal pups with different genotypes. As shown in S1C Fig., in these purer SMC cultures, expression of a major 130 kDa NRP1-immunoreactive band was reduced in *Nrp1^+/−^* heterozygotes compared with WT *Nrp1^fl/+^* mice, and was more strongly decreased in SMC from *Nrp1^SMKO^* mice. In whole colon extracts prepared from the same pups, western blots similarly showed a clear reduction in NRP1 expression in *Nrp1^SMKO^* mice (S1C Fig.). Additionally, we examined NRP1 expression in primary cultures of human colonic SMCs. NRP1-immunoreactive bands of 130kDa typical of full-length NRP1 as well as a higher molecular weight band of ∼250 kDa corresponding to glycosylated NRP1 and previously reported in human vascular SMC [12], were readily detected in human colonic SMCs, and their specificity was confirmed by effective knockdown using NRP1-targeted siRNA (S1D Fig.). These results indicated that NRP1 is expressed in gastrointestinal SMC in both neonatal and adult mice, and also demonstrated that we had generated a model of SMC-specific *Nrp1* deficiency. In all subsequent analyses, mice with SMC-specific *Nrp1* deficiency combined with global heterozygous loss of *Nrp1* (*smMHC-Cre^+/−^;Nrp1^fl/−^*, designated *Nrp1^SMKO^*) were compared with global *Nrp1* heterozygotes (*smMHC-Cre^−/−^;Nrp1^fl/−^*, designated *Nrp1^+/−^*), with additional comparisons with WT *Nrp1^fl/+^* mice (*smMHC-Cre^−/−^;Nrp1^fl/+^*, designated *Nrp1^fl/+^*). See “Materials and Methods” for full descriptions of the mouse genotypes used.

### *Nrp1^SMKO^* mice display a reduced rate of gastric emptying and develop lower GI tract abnormalities

**Figure 3:**
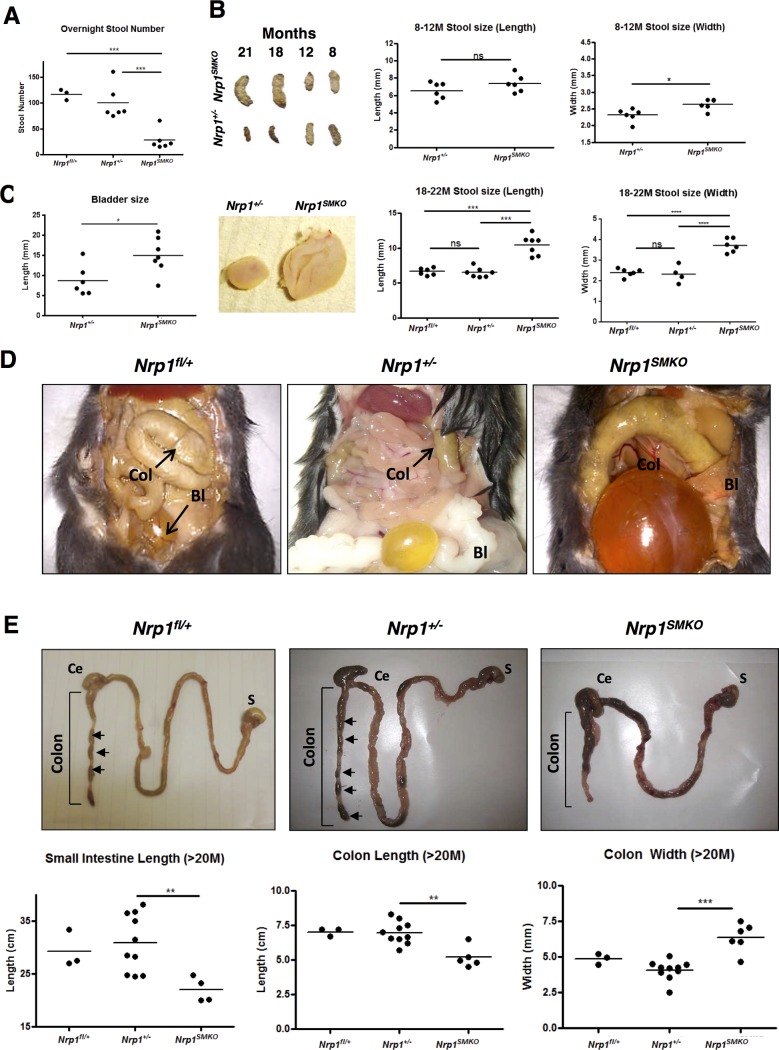
SMC-specific knockout of *Nrp1* causes severe constipation and GI tract malformations. (A) Overnight stool counts from ≥18 month-old mice. Mice were housed in a clean cage 24 hours before harvesting the stools. Differences between *Nrp1^SMKO^* and the controls were significant (***P<.001, n≥3). (B) Stool size measurement from 8–12 month- and 18–22 months-old mice. Each dot represents an average of six stools collected from one animal. (****P<.0001, ***P<.001, *P = .01). (C) Bladder length comparison between ≥18 month-old *Nrp1^SMKO^* and the controls, *P = .0217, n≥6. (D) Macroscopic images of dissected *Nrp1^fl/+^*, *Nrp1^+/−^* and *Nrp1^SMKO^*(≥18 months). The colon (Col) and the bladder (Bl) were severely dilated in *Nrp1^SMKO^*. (E) The entire GI tract was dissected from ≥20 months-old *Nrp1^fl/+^*, *Nrp1^+/−^* and *Nrp1^SMKO^* mice. The arrows point to well-pelleted faeces in the colon of the *Nrp1^fl/+^* and *Nrp1^+/−^* controls, while *Nrp1^SMKO^* displayed an impacted colon and caecum (Ce). The length of the colon and small intestine was significantly shorter in the *Nrp1^SMKO^* than the controls (**P<.01, n≥3). The width of the proximal colon was also significantly larger in the *Nrp1^SMKO^* compared to the controls (***P<.0001, n≥3).

*Nrp1^SMKO^* mutants were viable and fertile and appeared to develop normally and undergo normal post-natal growth. No overt vascular abnormalities were observed in adults either in large vessels, such as the aorta, or in the adult retinal vasculature (results not shown). However, by 18 months of age *Nrp1^SMKO^* mice exhibited abdominal distension and a significantly reduced rate of gastric emptying, indicated by reduced overnight stool count (Fig. 3*A*), larger stools with a significantly greater mean weight, and significantly reduced total overnight stool weight compared to their *Nrp1^+/−^* controls and WT *Nrp1^fl/+^* mice (Fig. 3B, S2A-B Fig.). At 5–8 months, stool width and length showed no significant differences in the *Nrp1^SMKO^* mutants compared to the heterozygous controls (S2C Fig.), whereas in 8–12 month old mice stool width was significantly greater in the *Nrp1^SMKO^* mutants (Fig. 3B). The difference in size became more pronounced with increasing age, and by 18–22 months of age stool length (*P*<.0001) and width (*P*<.0009) mice were significantly greater in *Nrp1^+/−;SMKO^* compared to *Nrp1^+/−^* mice (Fig. 3*C*). We did not observe any detectable significant differences in the total weights of older *Nrp1^SMKO^* compared to *Nrp1^+/−^* mice or WT *Nrp1^fl/+^* mice (S2D Fig.).

Examination of the GI tract in ≥18 month-old mice revealed a grossly enlarged colon in the *Nrp1^SMKO^* mutants, as well as a significantly distended bladder (Fig. 3*C-D*). In contrast, control *Nrp1^+/−^* and WT *Nrp1^fl/+^* mice showed normal colon and bladder of healthy appearance. Further macroscopic examination of *Nrp1^SMKO^* mice revealed a significantly shorter small intestine and colon as well as a significantly dilated colon, in which the stool had become impacted and hardened towards the rectum leading to severe constipation (Fig. 3*E*). In comparison, the colons of the controls contained stool that had formed into pellets as it reached the rectum (Fig. 3*E*).

**Figure 4:**
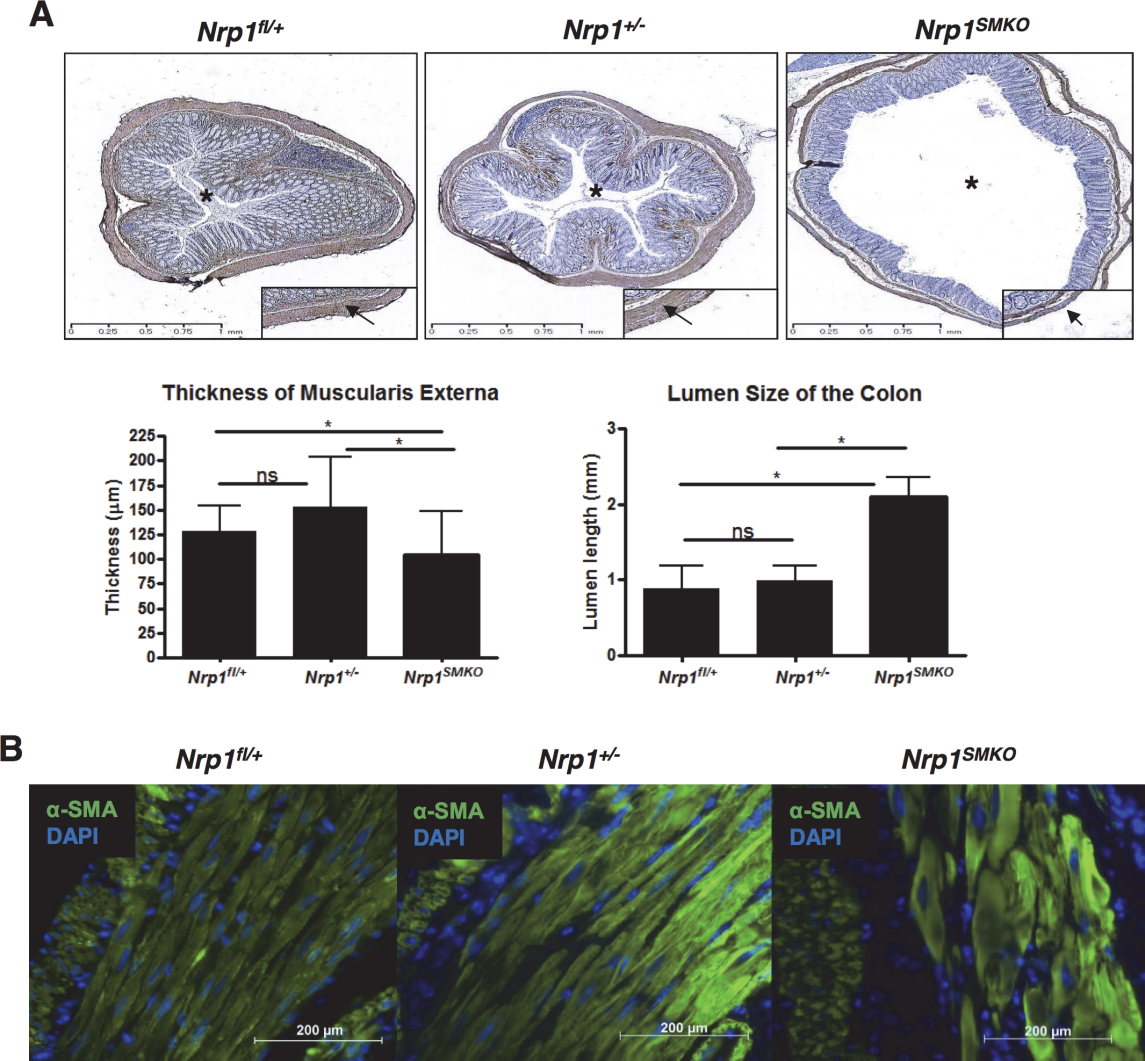
SMC-specific knockout of *Nrp1* leads to defects in smooth muscle morphology. (A) SMA immunohistochemistry on cross-sections of adult colon (≥18 months). Arrow points to the muscularis externa, which was significantly thinner in *Nrp1^SMKO^* (*P<0.05, *Nrp1^fl/+^* n = 4; *Nrp1^+/−^* n = 5; *Nrp1^SMKO^* n = 5). The lumen size (asterisk) was significantly larger in the *Nrp1^SMKO^*: means±SD, *P<0.05, *Nrp1^fl/+^* n = 3; *Nrp1^+/−^* n = 4; *Nrp1^SMKO^* n = 4. (B) SMA immunofluorescent staining of colonic tissue sections.

To examine whether Nrp1 deficiency affected the GI smooth muscle architecture and organisation colonic tissue sections from ≥18 month-old *Nrp1^SMKO^* mutants and their controls were collected and stained for α-SMA. Analysis of similar regions of the colon in *Nrp1^SMKO^* and heterozygous *Nrp1^+/−^* mice that were not filled with faeces, a method similar to that reported in other studies [29], revealed a significantly enlarged lumen size in the *Nrp1^SMKO^* mutants, consistent with the grossly enlarged colon observed upon dissection, as well as a complete lack of transverse folds that are normally supported by the muscularis mucosae and a significant thinning of the SMC layer in the muscularis externa (Fig. 4*A*). Immunofluorescent staining showed that SMC in the colons of *Nrp1^SMKO^* mice had a less elongated shape and were less tightly organised compared with heterozygous *Nrp1^+/−^* mice, characteristic of SMC that are hypertrophic (Fig. 4*B*). Examination of SMC associated with colonic submucosal blood vessels indicated that both the architecture of these vessels, the SMC content, and the number of vessels were grossly normal in *Nrp1^SMKO^* mice as compared with *Nrp1^+/−^* heterozygotes and WT *Nrp1^fl/+^* mice (results not shown).

We also investigated the organisation of enteric neurons in *Nrp1^SMKO^* mutants by staining for Class III beta-tubulin, encoded by the *Tubb3* gene, using an antibody that specifically recognises neurons in the adult mouse intestine [30,31]. TUBB3 staining of *Nrp1^fl/+^* and *Nrp1^+/−^* colon sections revealed clusters of neurons at the interface between circular and longitudinal smooth muscle layers, and neurons interspersed within the circular smooth muscle. TUBB3-positive neurons in *Nrp1^SMKO^* mice had a flattened and elongated appearance (S3*A* Fig.). Examination of *Tubb3* mRNA and TUBB3 protein expression showed no significant decrease in expression in *Nrp1^SMKO^* as compared to heterozygous *Nrp1^+/−^* mice (S3*B* and *C* Fig.). Furthermore, determination of the expression of the pan neuron-specific markers, *HuC* and *HuD*, using qPCR also revealed no specific differences between *Nrp1^SMKO^* as compared to heterozygous *Nrp1^+/−^* mice (S3D-E Fig.).

### Loss of *Nrp1* in SMC reduces GI smooth muscle contractility

**Figure 5:**
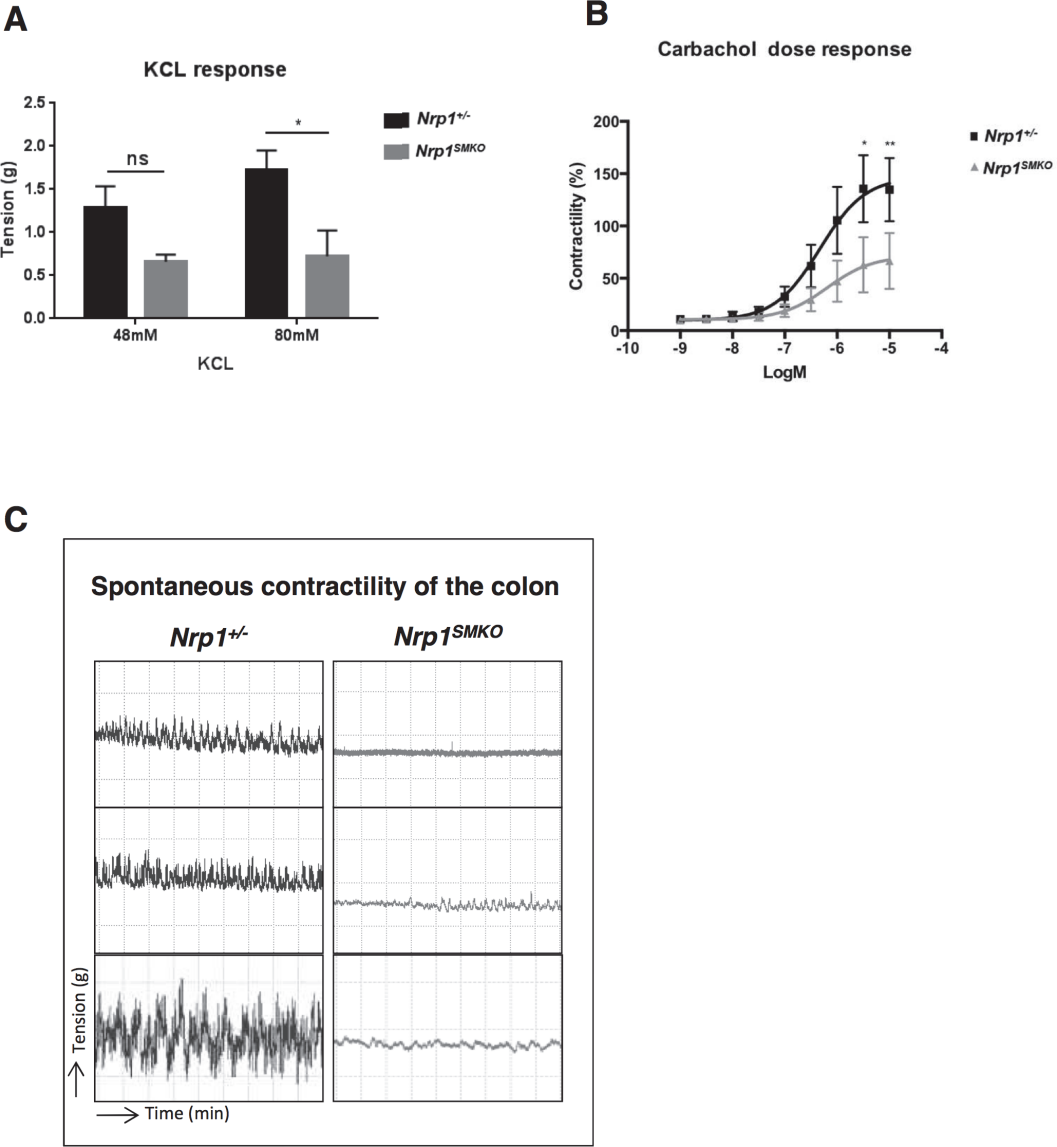
Decreased colonic contractility in *Nrp1^SMKO^*. Organ bath experiments showing the colonic contractile response to increasing doses of KCl (A) and CCh (B) in *Nrp1^SMKO^* and *Nrp1^+/−^* mice (means±SEM). A significant difference in the contractile tension in response to KCl was observed at 80mM (A, *P = .0359, n = 3). (B) Colonic segments of *Nrp1^SMKO^* had a significantly reduced contractility in response to CCh at 3μM (*P<.05, **P = .0088, n = 4). (C) Representative spontaneous contractions are shown for colonic segments from three different *Nrp1^SMKO^* and *Nrp1^+/−^* littermates.

The *Nrp1^SMKO^* mutants exhibit characteristics of severe gut dysmotility resulting in weakened peristaltic propulsion along the gut and indicating reduced or defective contractility. *Ex vivo* analysis of colonic ring contraction in response to KCl and the cholinergic agonist, CCh, revealed reduced contractile responses in the *Nrp1^SMKO^* mutants compared with controls (Fig. 5). A significant difference in the mean contractile tension during KCl depolarisation between *Nrp1^SMKO^* and heterozygous *Nrp1^+/−^* mice (≥18 month-old) was observed at 80mM KCl (*Nrp1^SMKO^*: .7±g, *Nrp1^+/−^*: 1.5±g, *P*<.005) at which the maximal contraction was induced (data not shown), and a trend towards a decrease was observed at 48 mM KCl (Fig. 5A). The contractile response to CCh was also significantly reduced in the *Nrp1^SMKO^* mutants compared to *Nrp1^+/−^* controls at 3μM (Fig. 5B; *Nrp1^SMKO^*: EC50 5.971e^−.07^, *Nrp1^+/−^*: EC50 4.085e^−.07^, **P<.01). Furthermore, the amplitude of spontaneous contractile activity from the *Nrp1^SMKO^* mutants was reduced when compared to that of heterozygous *Nrp1^+/−^* controls (Fig. 5C). The spontaneous contractility of colonic rings from the heterozygous *Nrp1^+/−^* controls was similar to that of WT *Nrp1^fl/+^* mice (S4 Fig.).

### Occurrence of an immature SMC phenotype in early post-natal *Nrp1^+/−;SMKO^* mice

We hypothesised that the impaired GI motility and contractility observed in adult *Nrp1^SMKO^* mice might result from phenotypic changes in SMC due to loss of *Nrp1* which had developed at an earlier stage of development. To determine when changes in smooth muscle began to occur in *Nrp1^SMKO^* mutants we measured the lengths of the colon and small intestine in postnatal day (P)7 pups and 6 month-old mice. No significant differences were observed in the length of the small intestine or colon in the *Nrp1^SMKO^* mutants compared to their littermate *Nrp1^+/−^* controls at P7 (S5*A* Fig.). However, at 6 months of age the lengths of the small intestine and colon were significantly reduced in *Nrp1^SMKO^* mice, though no significant change in colon width was detected at that age (S5*B* Fig.). These results pointed to earlier defects in the structure of the GI tract in *Nrp1^SMKO^* mice, which progressively worsened over time resulting in the severe functional GI defects observed in later adult life.

**Figure 6:**
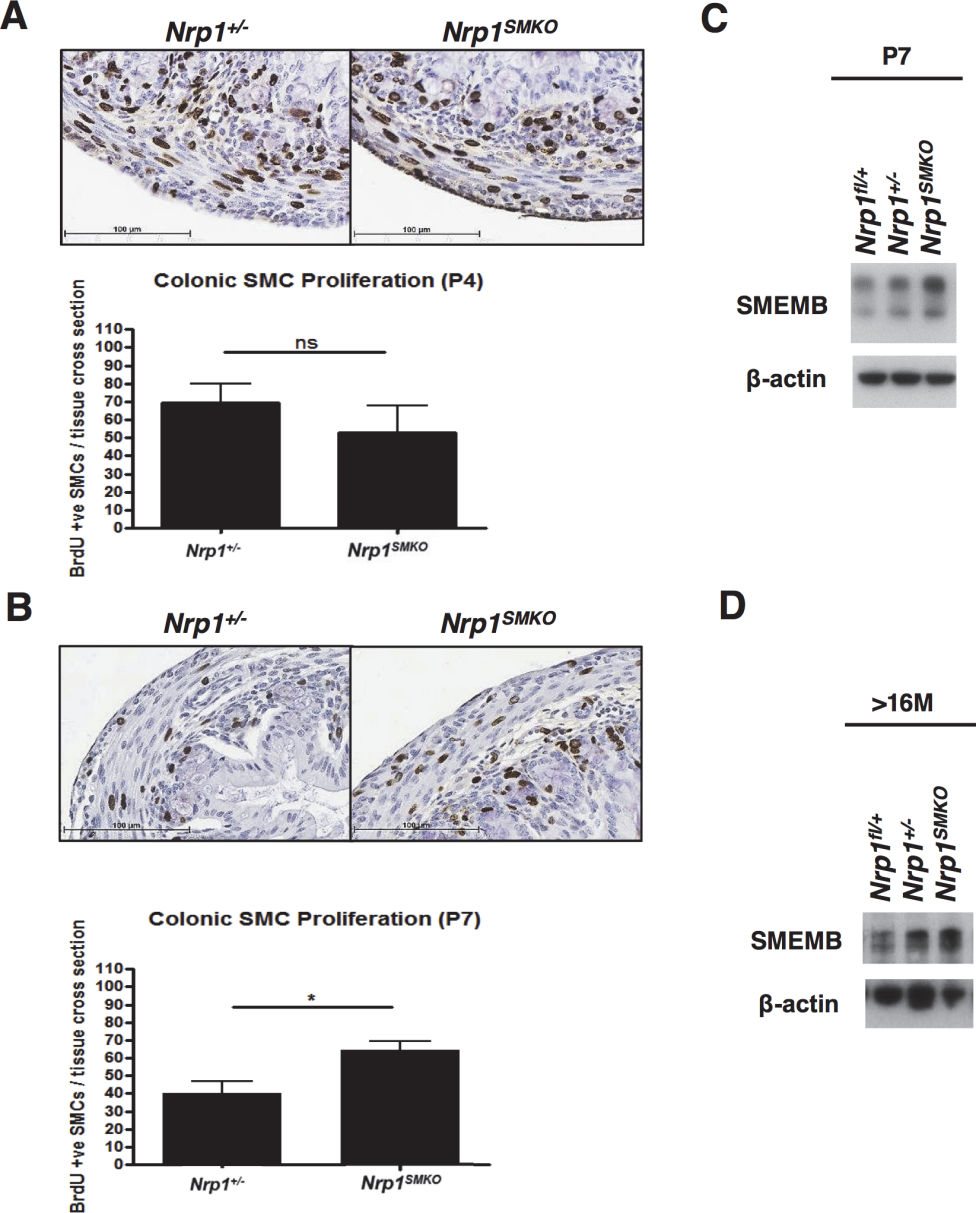
SMC proliferation and expression of SMEMB. (A) BrdU staining of colonic tissue sections at P4 (means±SD, n≥3). (B) Analysis of proliferation at P7 revealed a significant increase in SMC proliferation in *Nrp1^SMKO^* compared to the littermate controls (means±SD, *P = .0146, n = 3). (C,D) Western blots of SMEMB in bladder tissue extracts from P7 pups (C) and colonic extracts from adult (≥16 months, D) mice (n≥3).

The GI tract continues to develop after birth, as it adapts to changes in the gut microbiota and food composition at weaning, and reaches maturity in adulthood [32,33]. During GI development, SMC initially possess a less differentiated or synthetic phenotype characterised by a greater proliferative potential, and subsequently differentiate to acquire a contractile phenotype as the intestine matures into adulthood [34]. The defects we observed in *Nrp1^SMKO^* mice implied a possible defect in SMC differentiation which had long term consequences for SMC contractility. To investigate early changes in the phenotype of GI tract SMC we analysed both SMC proliferation and the expression of well-characterised protein markers of synthetic and contractile SMC in early postnatal pups. At P4 the proliferation of colonic SMC, assessed by *in vivo* BrdU labelling, was similar between *Nrp1^SMKO^* and *Nrp1^+/−^* mice (Fig. 6*A*). However, at P7 a significant increase in the number of proliferating SMC was detected in the *Nrp1^SMKO^* mutants compared to the controls, with SMC proliferation in *Nrp1^SMKO^* mice remaining at a level similar to that observed at P4, whereas proliferating SMC had decreased in the controls (Fig. 6*B*). We next quantified the colonic protein expression of the non-muscle myosin heavy chain B, SMEMB, a marker of de-differentiated or synthetic SMC [34]. Western blotting of bladder tissue extracts revealed a significant increase in SMEMB expression in P7 *Nrp1^SMKO^* pups as compared to their P7 globally heterozygous *Nrp1^+/−^* littermates (Fig. 6*C*, S6*A* Fig.). We also observed an increase in SMEMB expression in colonic tissue extracts from late adult (≥16 months-old) *Nrp1^SMKO^* mice (Fig. 6*D*), though this increase was not statistically significant (S6B Fig.). No significant differences were detected in colonic SMC proliferation in adult mice, and it was noted that both adult heterozygous *Nrp1^+/−^* control mice and *Nrp1^SMKO^* mutants possessed no or very few proliferating colonic SMC, though abundant proliferating cells were detected in the epithelial layers of the colon (S7 Fig.). These results point to a delay in the maturation/differentiation of GI tract SMC and persistence of a synthetic and proliferative SMC phenotype in *Nrp1^SMKO^* mice.

### Reduced expression of contractile SMC markers in *Nrp1^+/−;SMKO^* mice

**Figure 7:**
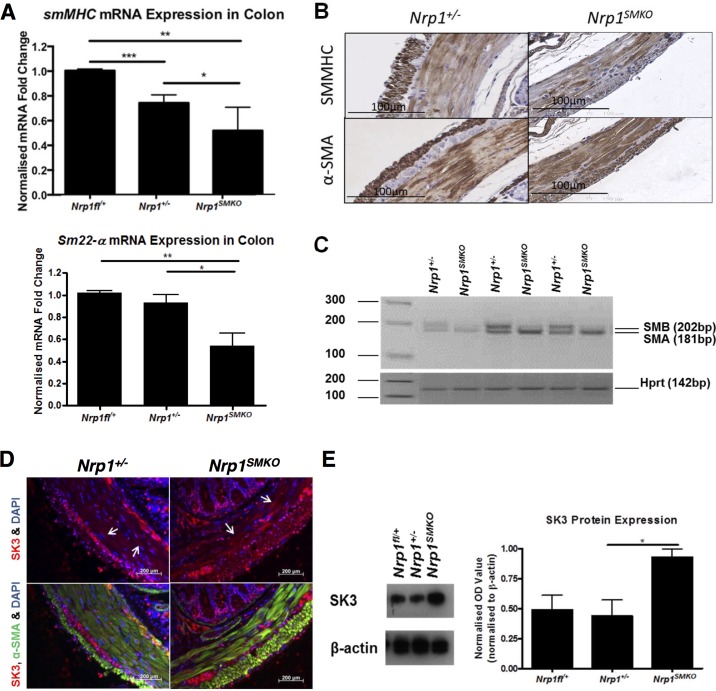
Loss of *Nrp1* in SMC affects the expression of markers of contractile phenotype. (A) qPCR analysis of SMC contractile phenotype markers, *smMHC* (n = 5) and *Sm22-alpha* (n≥3), on colonic samples from mice ≥16 months old: means±SD; *P<.05, **P<.005, ***P = .0005. (B) smMHC/SMA immunohistochemistry in colonic tissue from ≥16 months old mice. (C) Semi-quantitative RT-PCR for the SMA and SMB *smMHC* isoforms in whole colon cDNA from ≥16 month-old mice. Representative results are shown from 3 mice (*Nrp1^fl/+^*, n = 4; *Nrp1^+/−^*, n = 6; *Nrp1^SMKO^*, n = 6; *P = .04). (D) Immunofluorescent staining of SK3 and SMA in colons of ≥16 months old mice shows enhanced expression of SK3 in the colonic SMC (arrows) of *Nrp1^SMKO^* compared to *Nrp1^+/−^* controls. (E) Western blot of SK3 expression in colon lysates from ≥16 month-old mice. Densitometric quantification of SK3 was normalised to beta-actin expression: means±SD; *P = .0233, n = 4.

The delay in SMC differentiation and persistence of a more immature SMC phenotype observed in P7 *Nrp1^SMKO^* mutants, raised the possibility that a less differentiated SMC phenotype was maintained into later adult life, and could thereby explain the long-term effects of *Nrp1* SMC-specific deficiency on GI motility. We therefore examined the expression of markers of both contractile/differentiated and synthetic/de-differentiated SMC phenotypes in greater detail in adult mice (16–22M). Two widely studied markers of contractile SMC are smMHC and Sm22alpha [35]. Q-PCR analysis of *smMHC* and *Sm22alpha* mRNA expression levels in colonic tissue extracts revealed a significant reduction of both contractile SMC markers in the *Nrp1^+/−;SMKO^* mutants (*SmMHC* expression levels: *Nrp1^fl/+^* 99.6% ± 0.89%; *Nrp1^+/−^* 73.96% ± 8.79%; *Nrp1^SMKO^* 52.16% ± 19.05%. *Sm22α* expression levels; *Nrp1^fl/+^* 102.1% ± 3.6%; *Nrp1^+/−^* 93.38% ± 14.18%; *Nrp1^SMKO^* 54.19% ± 22.53%) (Fig. 7*A*). A reduction of smMHC protein expression in the colonic SMC of *Nrp1^SMKO^* mutants was also detected using immunohistochemical staining (Fig. 7*B*). In contrast expression of SMA, which is expressed at similar levels in synthetic and contractile SMC and does not differentiate between the two phenotypes, was unaffected in *Nrp1^SMKO^* mice.

We next investigated the relative expression of the SMA and SMB smMHC isoforms. The SMB isoform has higher ATPase activity [36,37], and is expressed in phasic smooth muscles (intestinal SMC) but absent in tonic smooth muscles (VSMC) [38]. We examined colonic SMA and SMB isoform expression from ≥16 month-old mice by PCR. We detected expression of the SMA isoform in all the samples analysed. However, whilst unambiguous expression of the SMB isoform was observed in ≥50% of the heterozygous *Nrp1^+/−^* samples analysed, in contrast, no expression of the SMB isoform could be detected in any of the *Nrp1^SMKO^* samples (Fig. 7*C*).

Loss of contractility in *Nrp1^SMKO^* mutants might also be due in part to a perturbation of the mechanisms regulating vascular tone. SK3 is a small conductance calcium-activated potassium channel, important for inhibitory neurotransmission in GI SMC, and is expressed in both the circular and longitudinal SMC in the murine small intestine and proximal colon [39,40]. Several studies indicate that SK3 channels regulate muscle tone in vascular and visceral smooth muscle, and that SK inhibition results in enhanced vasoconstriction and visceral smooth muscle contraction [41,42]. To examine the expression of SK3 in the *Nrp1^SMKO^* mutants, we performed double immunofluorescence labelling of colonic tissue sections for SK3 and SMA in ≥16 month-old mice. We observed increased expression of SK3 in both the circular and longitudinal SMC of *Nrp1^SMKO^* mice compared with littermate heterozygous *Nrp1^+/−^* controls (Fig. 7*D*). Furthermore, western blotting of colonic tissue extracts detected a significant increase in SK3 protein expression in *Nrp1^SMKO^* as compared with heterozygous *Nrp1^+/−^* mice (approximately a 2 fold increase) (Fig. 7*E*). This increased expression of SK3 likely contributes to the motility dysfunction seen in the colons of the *Nrp1^+/−;SMKO^* mutants. Changes in SK3 expression could arise from an expansion of SMC co-expressing PDGFR-alpha due to PDGF-A driven cell proliferation and/or migration of these cells. This possibility was examined by determining the levels of colonic PDGFR-alpha using qPCR. The results showed no significant differences in the mRNA expression of PDGFR-alpha in *Nrp1^SMKO^* mutants as compared with WT *Nrp1^fl/+^* or heterozygous *Nrp1^+/−^* mice (S8 Fig.).

## Discussion

Recent findings indicate that NRP1 is expressed in VSMC, and mediates signaling and migratory responses in SMC to PDGF, raising the possibility that NRP1 plays an important role in physiological and pathophysiological roles of smooth muscle *in vivo*. To investigate such a role we generated mutant mice with a conditional SMC-specific deficiency of *Nrp1. Nrp1^SMKO^* mice were viable with no overt phenotype or significant mortality in development or in early post-natal life. However, aged mice with SMC-specific loss of *Nrp1* unexpectedly exhibited signs of severely impaired GI motility characterised by increased size and reduced frequency of stool, reduced contractility of the colon, enlargement of the bladder and a distended and shortened large and small intestine, defects that were associated with a marked reduction in the thickness of the muscularis externa smooth muscle layer of the colon. Collectively, these defects indicate an essential role of NRP1 in the SMC of the gut in maintaining both healthy GI function and the normal architecture of visceral smooth muscle in later adult life.

Analysis of markers of the contractile and synthetic/proliferative phenotypes indicated that loss of *Nrp1* in SMC resulted in a shift of gut SMC to a less contractile and more synthetic state. In particular, the reduced expression in *Nrp1^SMKO^* mice of smMHC and its SMB isoform is consistent with the reduced colonic contractility in these mutants. The SMB isoform is expressed mainly in bladder, intestine, stomach, and small muscular arteries, and is the predominant isoform in intestine [43]. Differential expression of SMA and SMB isoforms may be related to their different properties in actin-myosin force generation. Thus, SMB containing a 7 amino acid insert, exhibits higher rates of MgATP binding to and MgADP release from the myosin active site, allowing myosin to transition through the cross-bridge cycle at an increased rate [44]. This in turn may account for the greater actin filament velocity characteristic of tissues with predominantly phasic smooth muscle (e.g. the gut wall) as compared with tonic smooth muscle (e.g. most blood vessels). It is also noteworthy that down-regulation of SMB isoform expression has been reported in the lethal spotted mouse, a model of Hirschsprung’s disease, characterised by megacolon and functional obstruction of the colon proximal to the rectum [45], and in the hypertrophic urinary bladder [46]. The increased expression in *Nrp1^SMKO^* mutants of the small Ca^2+^-activated K^+^ channel, SK3, implicated in negative regulation of smooth muscle contraction, further indicates that mechanisms regulating smooth muscle contractility are disrupted in *Nrp1^SMKO^* mice. This conclusion is also supported by immunofluorescent staining of colonic smooth muscle showing that SMC in *Nrp1^SMKO^* mice had a less spindle-shaped appearance and were less closely arranged with larger intercellular spaces, characteristic of hypertrophic and more synthetic cells. Though mutant mice lacking *Nrp1* in SMC appeared outwardly normal during development and early post-natal and adult life, we observed increased SMC proliferation and expression of SMemb, a marker of the synthetic phenotype, in early post-natal *Nrp1^SMKO^* pups (P7). Furthermore, a significant reduction in the length of the small and large intestines was observed as early as 6 months in *Nrp1^SMKO^* mice. Together, these findings indicate that loss of *Nrp1* in gut SMC causes early post-natal changes in SMC resulting in a persistent loss of their contractile phenotype in turn contributing to progressively worsening defects in GI structure and late-onset impairment of GI contractility and motility. It is plausible that the increased proliferative capacity of SMC in *Nrp1^SMKO^* mice and other changes associated with the synthetic phenotype make intestinal smooth muscle more susceptible to long-term degenerative processes resulting in thinning of the intestinal smooth muscle layer and loss of function. Since gastrointestinal SMC express PDGFRα and its major ligand PDGF-A, changes in SK3 expression might be due to expansion of SMC or non-SMC co-expressing PDGFR-alpha due to PDGF-A driven cell proliferation and/or migration. Though we found no significant changes *Pdgfr-alpha* mRNA expression in *Nrp1^SMKO^* mutants as compared with WT *Nrp1^fl/+^* or globally heterozygous *Nrp1^+/−^* mice, it is plausible that PDGF-A signaling via PDGFRα may play a role in increasing SK3 expression, and in promoting a switch in gastrointestinal SMC to a more synthetic, less differentiated phenotype. This possibility warrants further study. Though we did not observe gross changes in the early post-natal GI tract, further investigation of the role of *Nrp1* in the early stages of GI development may be warranted. It is of interest that mice null for *Nrp2* exhibited enhanced bladder smooth muscle contractility [47]. We did not examine bladder contractility in our study, but the distension of the bladder observed in *Nrp1^SMKO^* mice is consistent with the other pathological GI features described herein. It is possible therefore that NRP1 and NRP2 may play different, even opposing, roles in regulating visceral smooth muscle function.

The viability and apparently normal development of *Nrp1^SMKO^* mice indicates that loss of *Nrp1* expression in SMC does not significantly impact upon cardiovascular development, angiogenesis, or arteriogenesis, though we do not preclude the possibility that targeted deletion of *Nrp1* in SMC may have more subtle or organ-specific effects on cardiovascular integrity and development. Furthermore, it is plausible that residual SMC NRP1 expression perhaps due to incomplete Cre-mediated deletion of *Nrp1* in our model is sufficient to perform some key functions of NRP1 during embryonic and post-natal development.

Chronic obstruction of the large bowel and severe constipation in humans can be caused by defects either in the enteric nervous system or in the smooth muscle. Hirschsprung’s disease, the main genetic cause of chronic life-endangering constipation with a frequency of 1:5000 live births, results in many cases from mutations in specific genes, such as *Ret* or *Gdnf*, implicated in differentiation or patterning of the enteric nervous system, giving rise to an aganglionic megacolon [48,49]. In contrast, the genetic or other causes of GI motility disorders of myogenic origin are largely unknown. While Hirschsprung’s disease usually presents in infancy, chronic idiopathic intestinal motility disorders of myogenic origin occur in adults, though rarely, and are associated with visceral myopathy, including atrophy and degeneration of the intestinal smooth muscle layers [50,51]. The aberrant features of the GI tract observed in *Nrp1^SMKO^* mice are similar, though less severe and later in onset, to the phenotype of mice with SMC-specific deletion of Serum Response Factor (SRF), a transcription factor for many contractile SMC genes [29]. *SRF^SMKO^* mice exhibited chronic intestinal pseudo-obstruction (CIPO) resulting in cachexia and death between P13 to P22, and associated with decreased intestinal length and dilation of the large bowel, thinning of the intestinal SMC layer and reduced intestinal contractility. Several of these defects are similar to those observed in *Nrp1^SMKO^* mice and in human CIPO. The loss of SMC-specific markers in intestinal SMC is associated with some forms of human GI motility dysfunction. For example, reduced jejunal α-SMA expression has been reported in idiopathic CIPO [52]. The role of NRP1 in human gastrointestinal SMC function is unknown, but we found strong expression of NRP1 in primary cultures of human colonic SMC (S1 Fig.). Our findings raise the possibility that loss of *Nrp1* in visceral smooth muscle could potentially be an underlying contributor to myopathy leading to impaired GI motility in human CIPO, a possibility that merits future investigation.

This is the first study to report the effects of *in vivo* loss of *Nrp1* in SMC, and the first to demonstrate a role of *Nrp1* in visceral SMC function *in vivo*. The finding that *Nrp1* is important for maintenance of adult mouse intestinal contractile smooth muscle function and for preservation of normal intestinal smooth muscle architecture identifies new roles for this molecule *in vivo*. Though we have not defined the precise mechanism involved, our results indicate a role for this molecule in promoting the sustained transition of visceral SMC to a more contractile phenotype from early post-natal life. This suggests that NRP1 mediates the actions of an extracellular factor or factors that promote differentiation of visceral SMC to the mature contractile state required for their functions in later adult life. Defining those differentiation factors that act via NRP1 in visceral SMC should be a focus of future research.

## Supporting Information

S1 Fig Expression of NRP1 in gastrointestinal SMC. A. Densitometry analysis shows a significant reduction in NRP1 protein expression in colonic tissue extracts from *Nrp1^SMKO^* adult mice (>18 months-old) compared to *Nrp1^+/−^* controls (*P = .041). B. NRP1 protein is expressed in colonic smooth muscle cells as detected by immunofluorescence staining of colonic tissue sections from wild-type adult (5–6 months-old) mice. C. Western blotting also confirmed expression of NRP1 in extracts from purified murine bladder smooth muscle cells and whole colonic tissue from postnatal day 7 pups, this expression was markedly reduced in tissue extracts from *Nrp1^+/−^* and *Nrp1^SMKO^* littermates. D. NRP1 protein is also expressed in human colonic smooth muscle cells and is efficiently knocked down following treatment of the cells with siNRP1.
(TIFF)

S2 Fig Effects of SMC-specific *Nrp1* knock out on stool weight, stool size and body weight. Mean stool weight was significantly larger in *Nrp1^SMKO^* mice compared to *Nrp1^+/−^* and *Nrp1^fl/+^* controls (A, means ±SD, n = 6, *P<.05) reflecting the larger stool size of these mice. Total weight of overnight stool (mean stool weight multiplied by total number of stools) in *Nrp1^+/−;SMKO^* mice was significantly reduced compared to *Nrp1^fl/+^* and *Nrp1^+/−^* controls (B, means ±SD, n≥4, *P<.05), reflecting the reduction in overnight stool number which is likely due to slow gastric motility. No significant difference in stool length or width (C) could be detected in *Nrp1^SMKO^* mice at 5–8 months of age (n = 4). No significant difference in body weight was detected in aged (>14 months-old) *Nrp1^SMKO^* mice compared to *Nrp1^+/−^* and *Nrp1^fl/+^* controls (D, means ±SD, n≥5).
(TIFF)

S3 Fig *Nrp1* deficiency in SMC did not significantly affect expression of neuronal markers. Class III beta-tubulin/TUBB3 immunostaining of colon sections revealed an aberrant staining pattern in *Nrp1^SMKO^* compared to controls (A), but *Nrp1* deficiency in SMC did not significantly affect *Tubb3* mRNA (B, means±SD, n = 4) and protein (C, n = 3) expression. Expression of the pan-neuronal markers *HuC* (D) and *HuD* (E) were also not significantly different between *Nrp1^fl/+^*, *Nrp1^+/−^* and *Nrp1^SMKO^* mice (16–22 months-old), as determined by Q-PCR of mRNA prepared from whole colon extracts.
(TIFF)

S4 Fig Spontaneous colonic contractility in WT *Nrp1^fl/+^*, *Nrp1^+/−^* and *Nrp1^SMKO^* mice (≥18 months-old). The amplitude of spontaneous contractile activity was measured in colonic ring segments from *Nrp1^fl/+^*. *Nrp1^+/−^* and *Nrp1^SMKO^* mice in organ bath experiments. The spontaneous contractile activity was similar between *Nrp1^fl/+^* and *Nrp1^+/−^* mice and markedly different from *Nrp1^SMKO^* mice.
(TIFF)

S5 Fig Intestinal length in P7 and 6 months-old mice. A) No changes in the length of small intestine (*P* = .3344, n = 3) and colon (*P* = .8058, n = 3) were detected in P7 *Nrp1^SMKO^* compared to their littermate controls. B) At 6 months of age *Nrp1^SMKO^* mutants began to display a significant shortening of the small intestine (***P<.0001, n≥6) and colon length (*P<.05, n≥5) compared to the littermate controls, but no significant changes in the colon width were detected (*P* = .4335, n≥6).
(TIFF)

S6 Fig Quantification by densitometry analysis of SMEMB protein expression. A. A significant increase in SMEMB expression was detected in bladder tissue extracts from P7 *Nrp1^SMKO^* neonates compared to their littermate controls (*P = .0255). B. A trend towards increased SMEMB expression was also detected in colonic tissue extracts from *Nrp1^SMKO^* adult mice (>16 months-old) compared to controls, however, this did not reach statistical significance.
(TIFF)

S7 Fig Proliferation of colonic SMC in adult *Nrp1^SMKO^* and control mice. BrdU staining of adult colonic tissue sections showed no significant difference in SMC proliferation between *Nrp1^SMKO^* and control littermates, *Nrp1^+/−^*.
(TIFF)

S8 Fig Colonic *Pdgfr-alpha* mRNA expression. No significant difference was seen in PDGFR-alpha mRNA levels by Q-PCR in colonic tissue extracts from *Nrp1^fl/+^*, *Nrp1^+/−^* and *Nrp1^SMKO^* 16–22 months-old mice (mean±SD, n≥3).
(TIFF)

S1 File Contains supporting Materials and Methods, Methods 1–5. (DOCX)

S1 Table Primers used for DNA genotyping. (TIFF)

S2 Table Antibodies used for Western Blotting analyses. (TIF)

S3 Table Antibodies for Immunohistochemistry. (TIF)

S4 Table qPCR and semi-quantitative RT-PCR primers. All primers work at 60°C annealing temperature.
(TIFF)

S5 Table Predicted and observed frequencies for each genotype. Predicted and observed frequencies for each genotype, expressed as percentage of the total number of animals genotyped (total number shown in parentheses). Four different genotypes were generated: *Nrp1^fl/+^*, *Nrp1^SMHET^*, *Nrp1^+/−^* and *Nrp1^SMKO^* close to the expected Mendelian ratio of 1:1:1:1.
(TIFF)
