# Supplementary figures and images for: Neuropilin 1 Is Essential for Gastrointestinal Smooth Muscle Contractility and Motility in Aged Mice
Source: PLoS One. 2015 Feb 6;10(2):e0115563. doi: 10.1371/journal.pone.0115563 (PMC4319892; doi:10.1371/journal.pone.0115563)

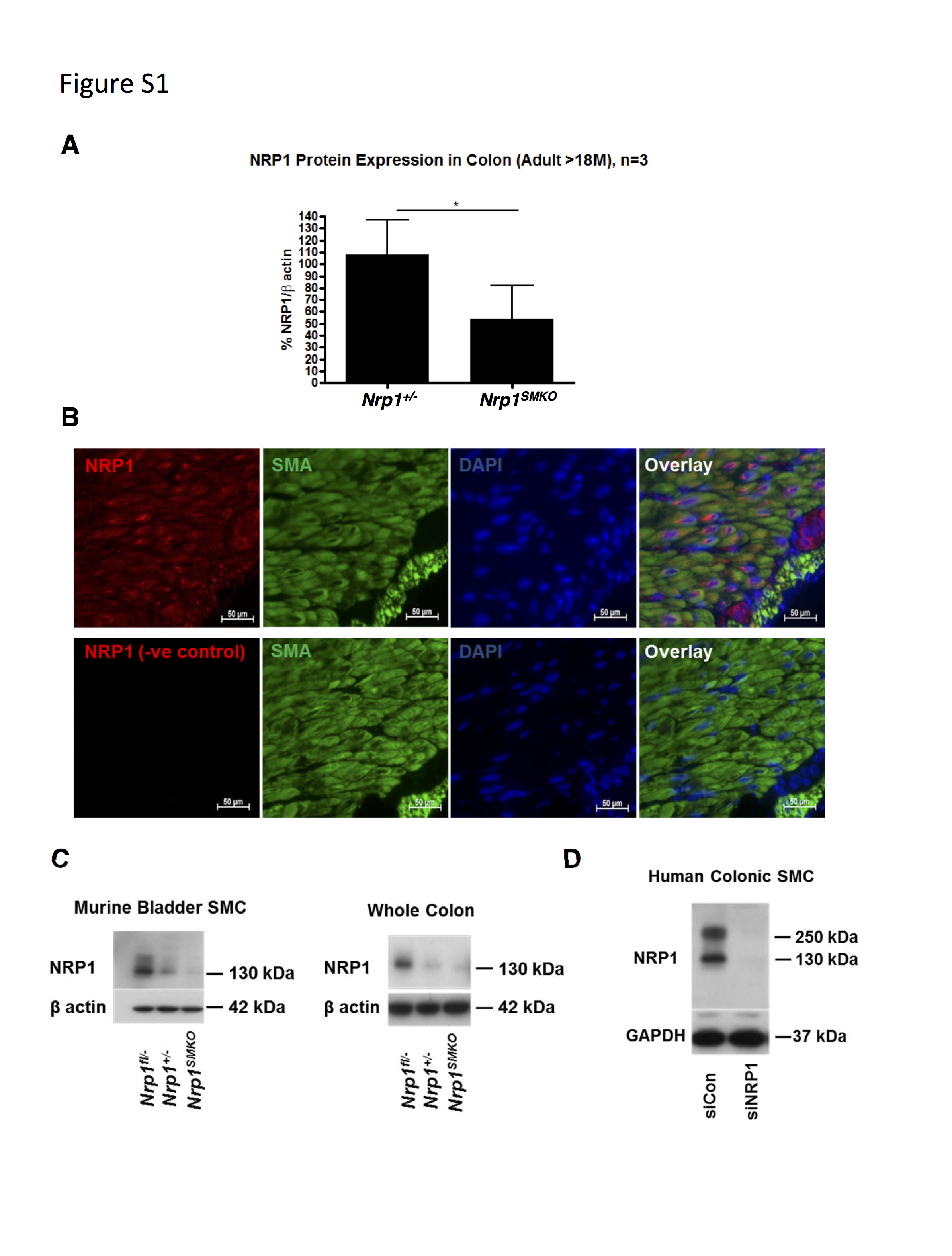

Supplement: S1 Fig — A. Densitometry analysis shows a significant reduction in NRP1 protein expression in colonic tissue extracts from Nrp1SMKO adult mice (>18 months-old) compared to Nrp1+/− controls (*P = .041). B. NRP1 protein is expressed in colonic smooth muscle cells as detected by immunofluorescence staining of colonic tissue sections from wild-type adult (5–6 months-old) mice. C. Western blotting also confirmed expression of NRP1 in extracts from purified murine bladder smooth muscle cells and whole colonic tissue from postnatal day 7 pups, this expression was markedly reduced in tissue extracts from Nrp1+/− and Nrp1SMKO littermates. D. NRP1 protein is also expressed in human colonic smooth muscle cells and is efficiently knocked down following treatment of the cells with siNRP1. (TIFF) [file pone.0115563.s001.tiff]

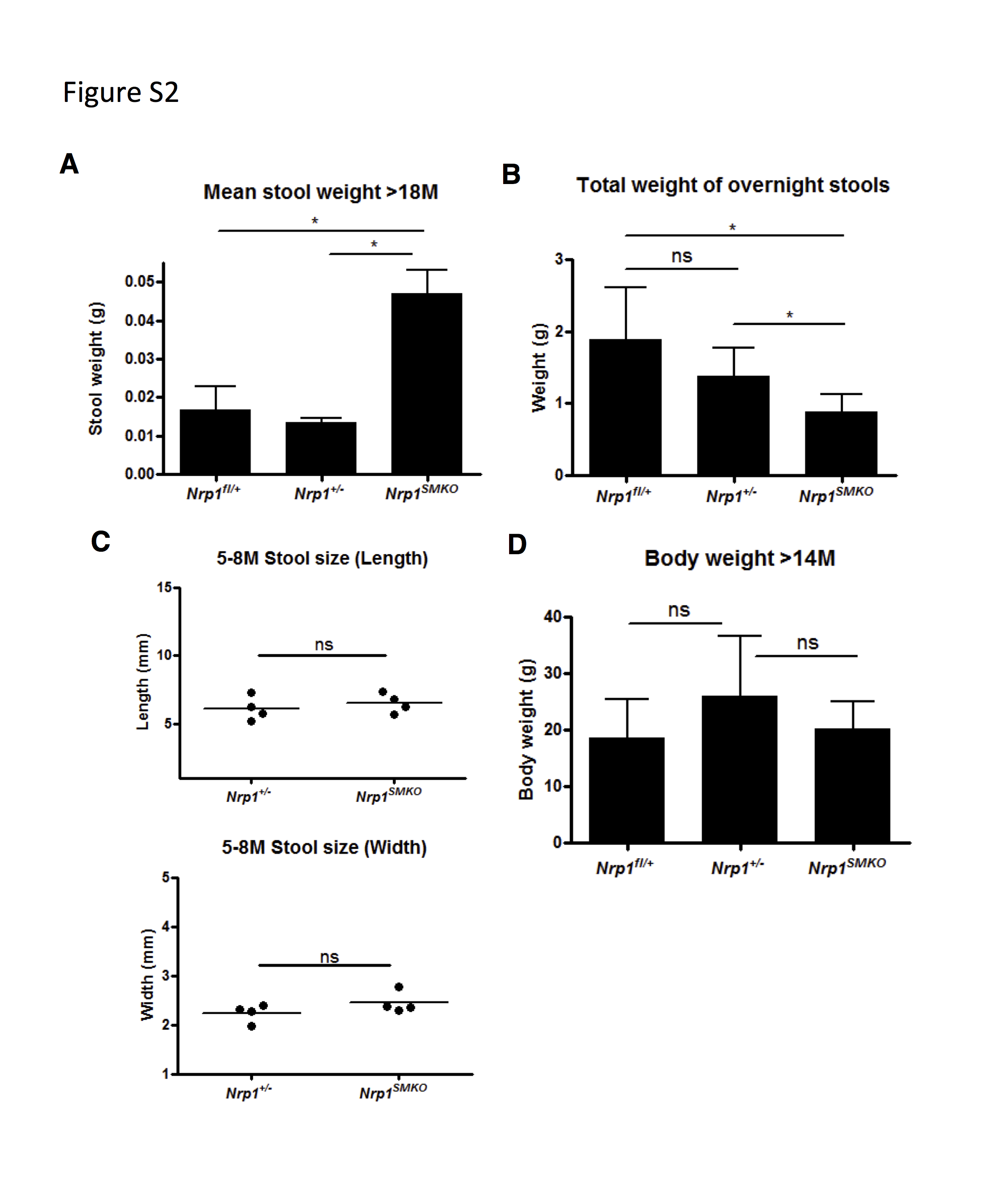

Supplement: S2 Fig — Mean stool weight was significantly larger in Nrp1SMKO mice compared to Nrp1+/− and Nrp1fl/+ controls (A, means ±SD, n = 6, *P<.05) reflecting the larger stool size of these mice. Total weight of overnight stool (mean stool weight multiplied by total number of stools) in Nrp1+/−;SMKO mice was significantly reduced compared to Nrp1fl/+ and Nrp1+/− controls (B, means ±SD, n≥4, *P<.05), reflecting the reduction in overnight stool number which is likely due to slow gastric motility. No significant difference in stool length or width (C) could be detected in Nrp1SMKO mice at 5–8 months of age (n = 4). No significant difference in body weight was detected in aged (>14 months-old) Nrp1SMKO mice compared to Nrp1+/− and Nrp1fl/+ controls (D, means ±SD, n≥5). (TIFF) [file pone.0115563.s002.tiff]

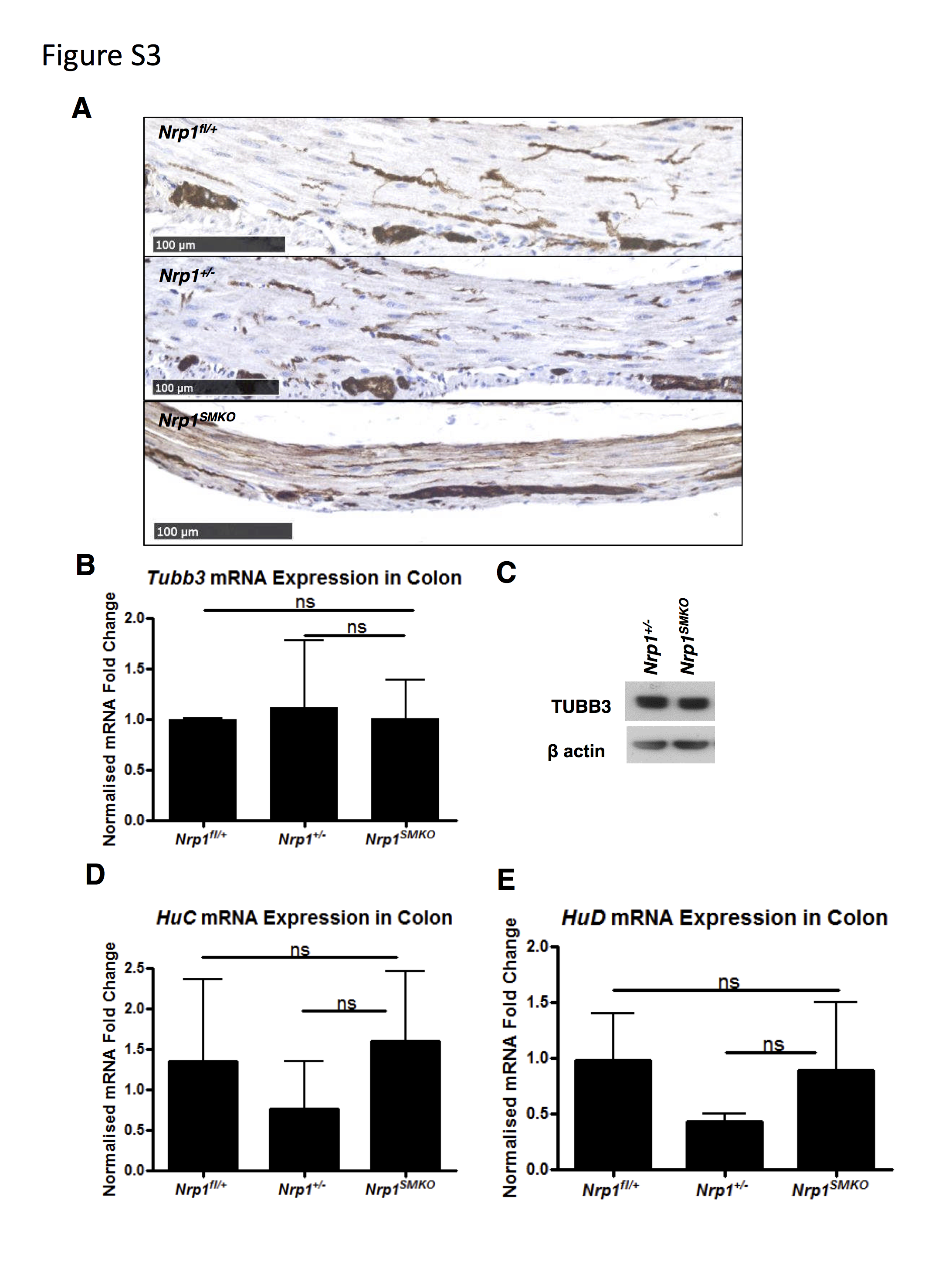

Supplement: S3 Fig — Class III beta-tubulin/TUBB3 immunostaining of colon sections revealed an aberrant staining pattern in Nrp1SMKO compared to controls (A), but Nrp1 deficiency in SMC did not significantly affect Tubb3 mRNA (B, means±SD, n = 4) and protein (C, n = 3) expression. Expression of the pan-neuronal markers HuC (D) and HuD (E) were also not significantly different between Nrp1fl/+, Nrp1+/− and Nrp1SMKO mice (16–22 months-old), as determined by Q-PCR of mRNA prepared from whole colon extracts. (TIFF) [file pone.0115563.s003.tiff]

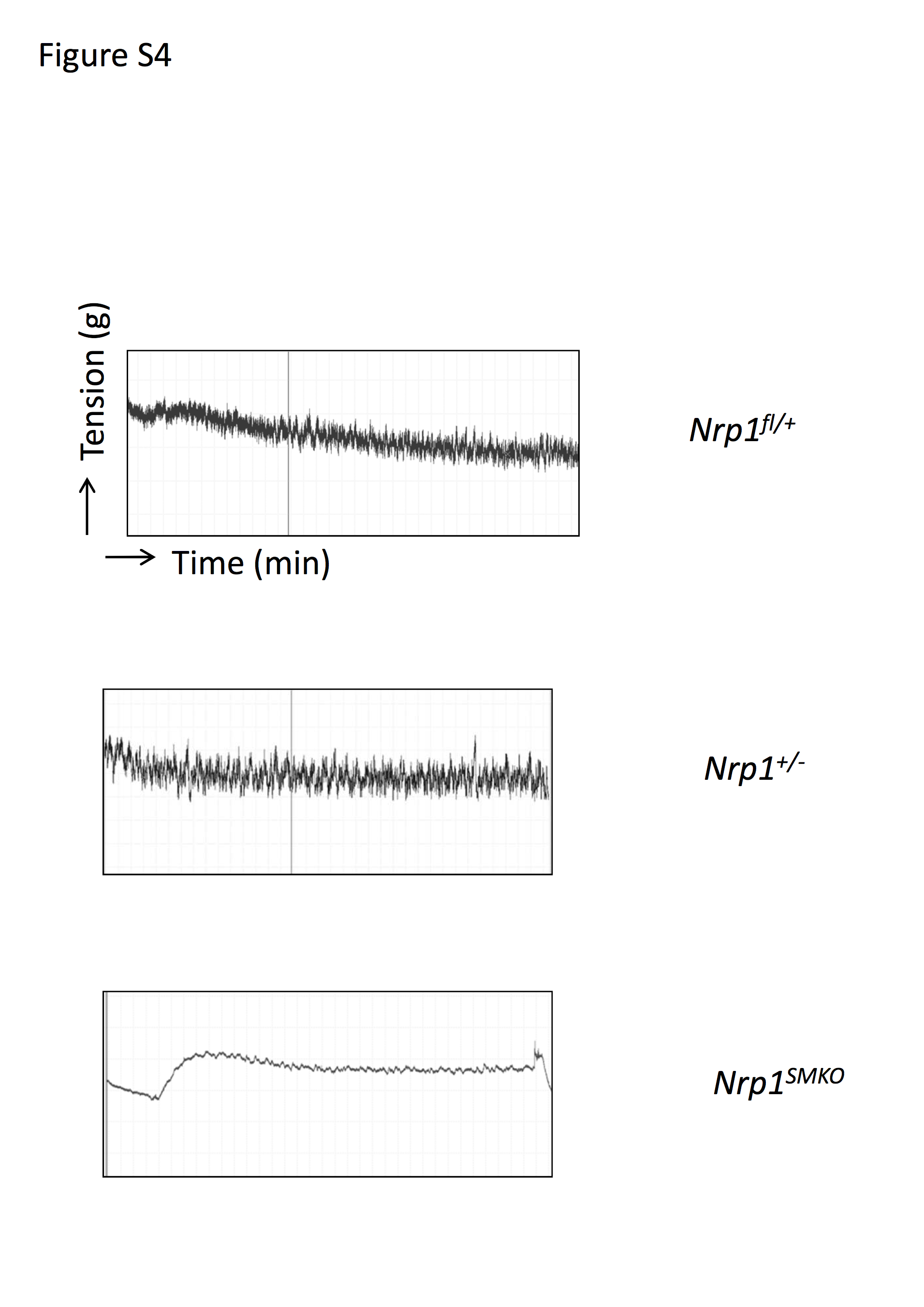

Supplement: S4 Fig — The amplitude of spontaneous contractile activity was measured in colonic ring segments from Nrp1fl/+. Nrp1+/− and Nrp1SMKO mice in organ bath experiments. The spontaneous contractile activity was similar between Nrp1fl/+ and Nrp1+/− mice and markedly different from Nrp1SMKO mice. (TIFF) [file pone.0115563.s004.tiff]

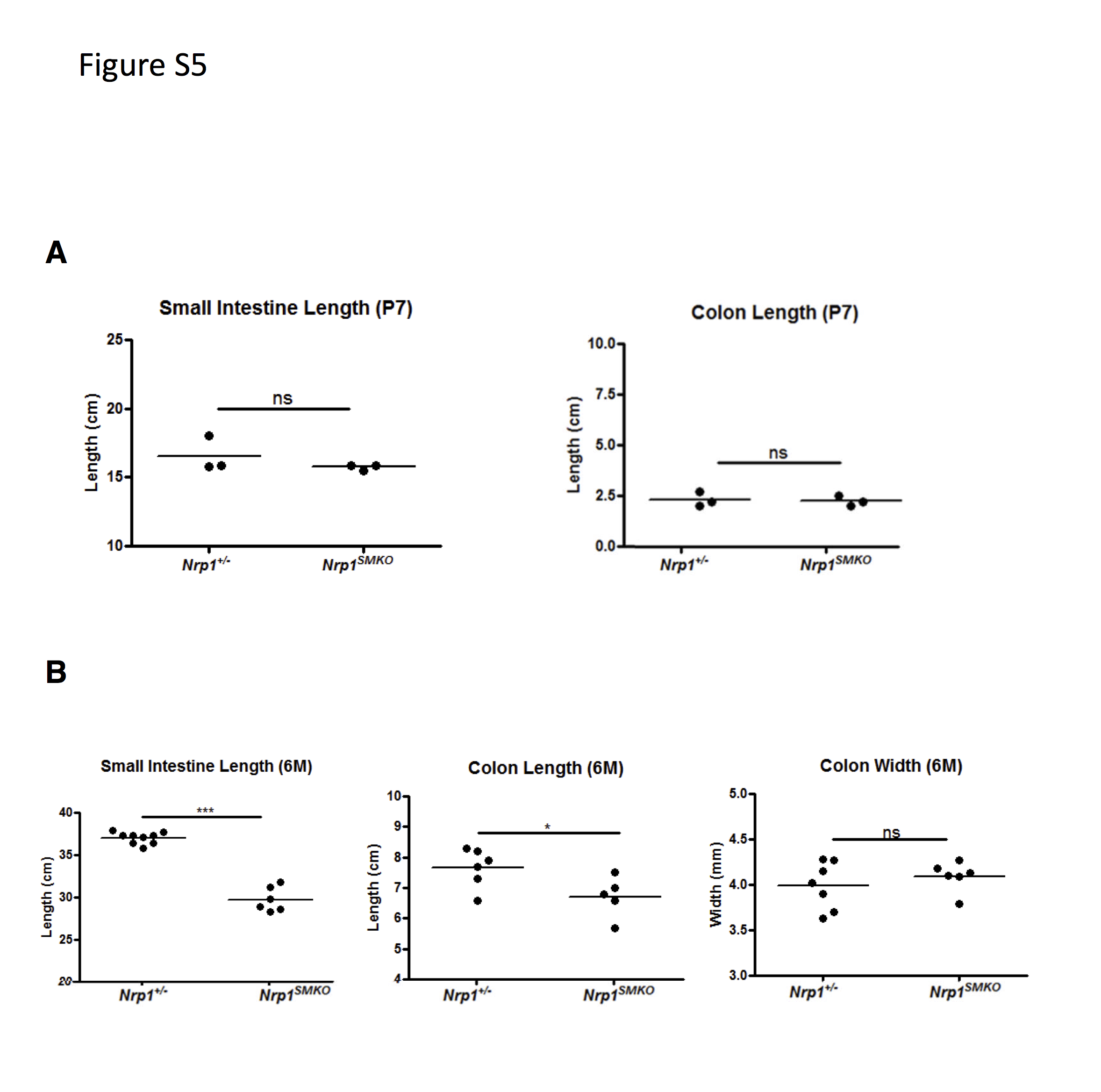

Supplement: S5 Fig — A) No changes in the length of small intestine (P = .3344, n = 3) and colon (P = .8058, n = 3) were detected in P7 Nrp1SMKO compared to their littermate controls. B) At 6 months of age Nrp1SMKO mutants began to display a significant shortening of the small intestine (***P<.0001, n≥6) and colon length (*P<.05, n≥5) compared to the littermate controls, but no significant changes in the colon width were detected (P = .4335, n≥6). (TIFF) [file pone.0115563.s005.tiff]

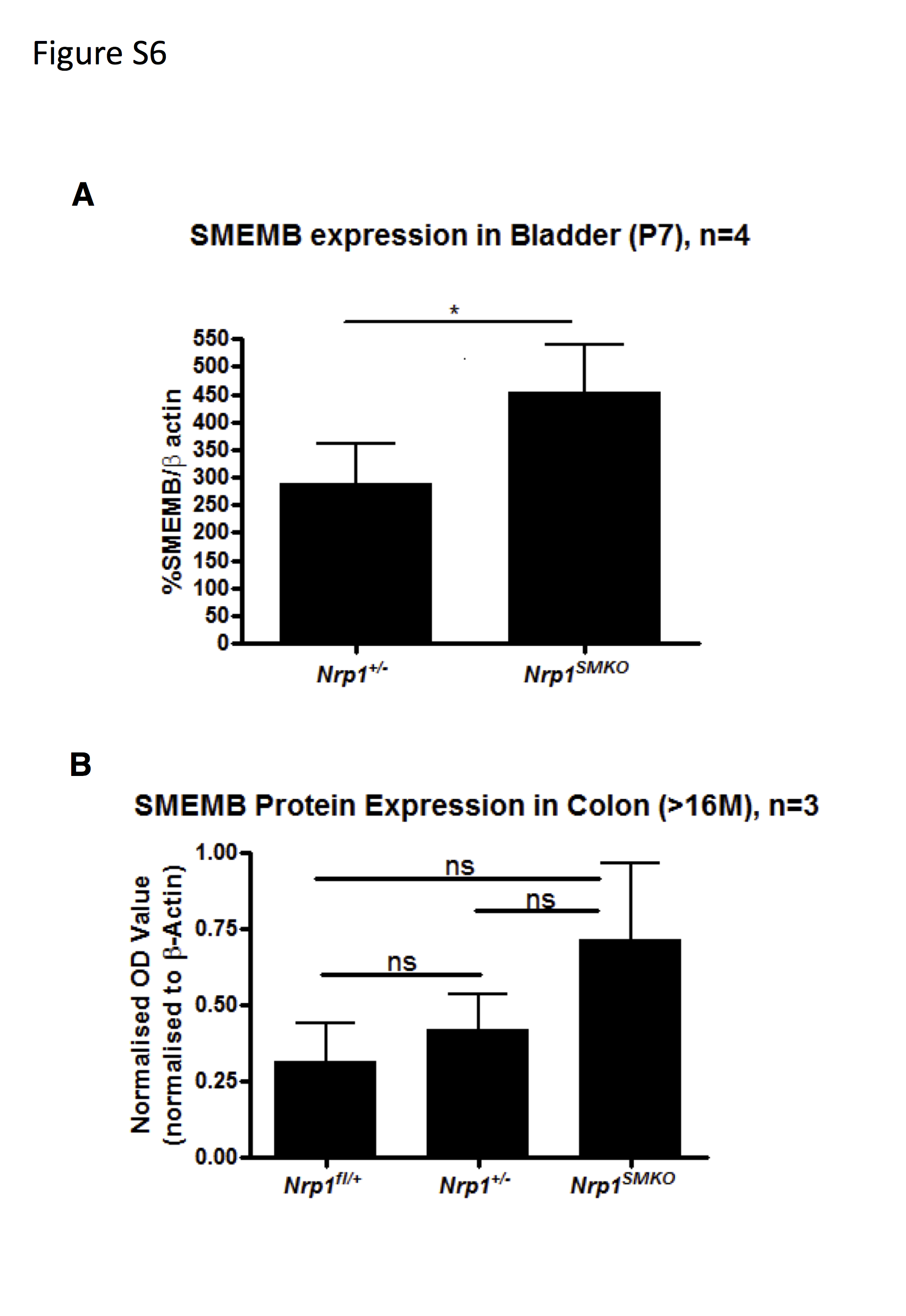

Supplement: S6 Fig — A. A significant increase in SMEMB expression was detected in bladder tissue extracts from P7 Nrp1SMKO neonates compared to their littermate controls (*P = .0255). B. A trend towards increased SMEMB expression was also detected in colonic tissue extracts from Nrp1SMKO adult mice (>16 months-old) compared to controls, however, this did not reach statistical significance. (TIFF) [file pone.0115563.s006.tiff]

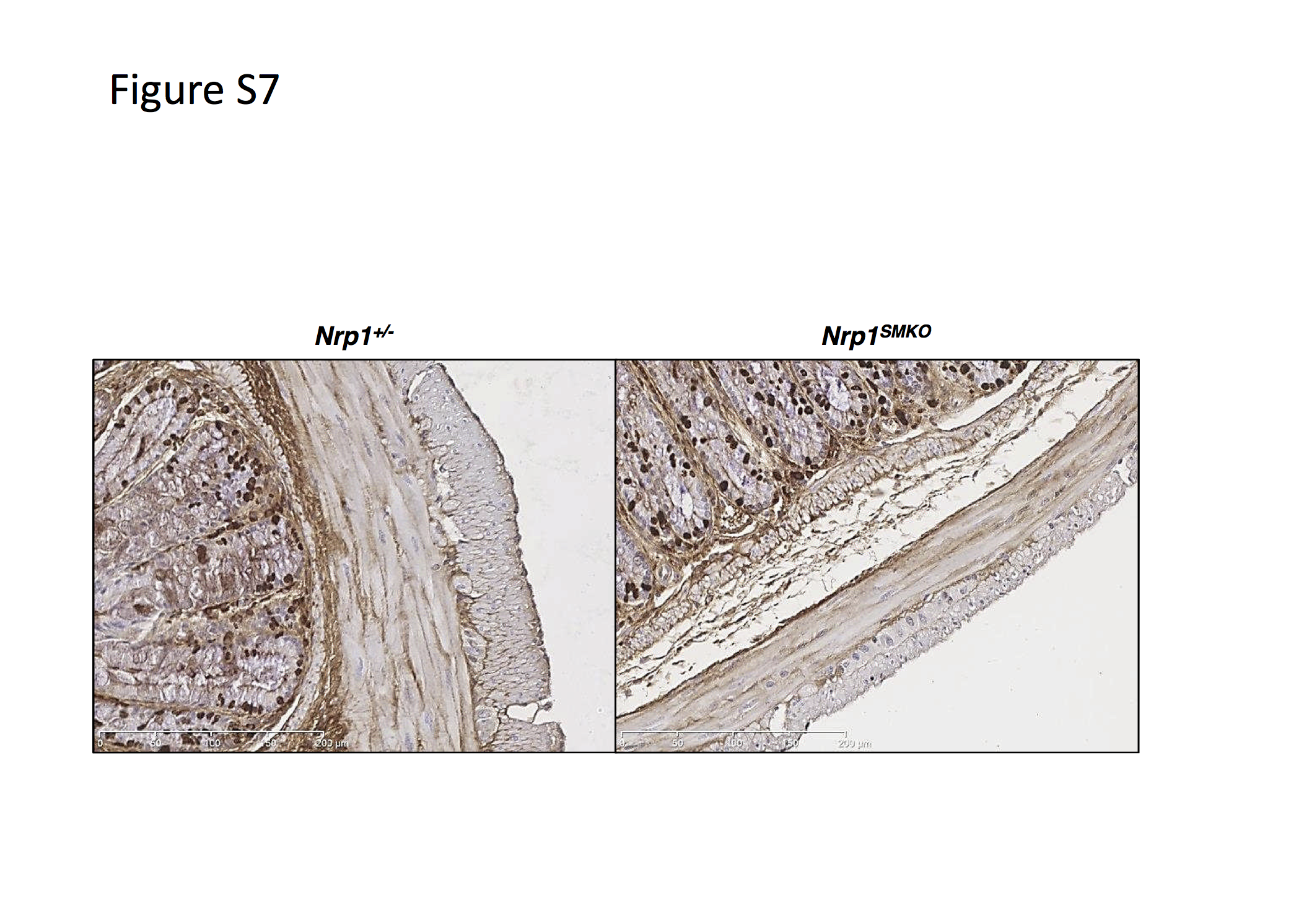

Supplement: S7 Fig — BrdU staining of adult colonic tissue sections showed no significant difference in SMC proliferation between Nrp1SMKO and control littermates, Nrp1+/−. (TIFF) [file pone.0115563.s007.tiff]

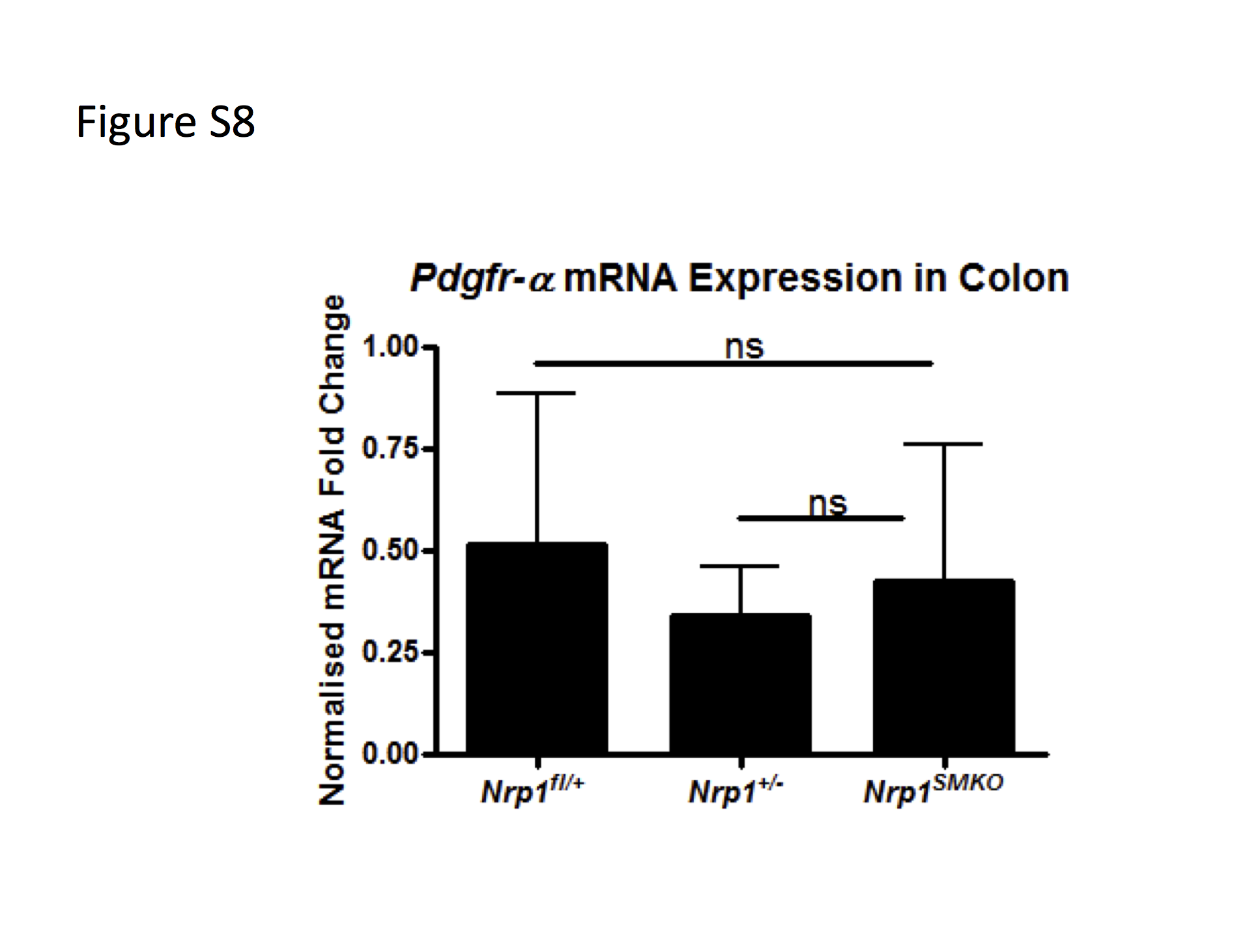

Supplement: S8 Fig — No significant difference was seen in PDGFR-alpha mRNA levels by Q-PCR in colonic tissue extracts from Nrp1fl/+, Nrp1+/− and Nrp1SMKO 16–22 months-old mice (mean±SD, n≥3). (TIFF) [file pone.0115563.s008.tiff]

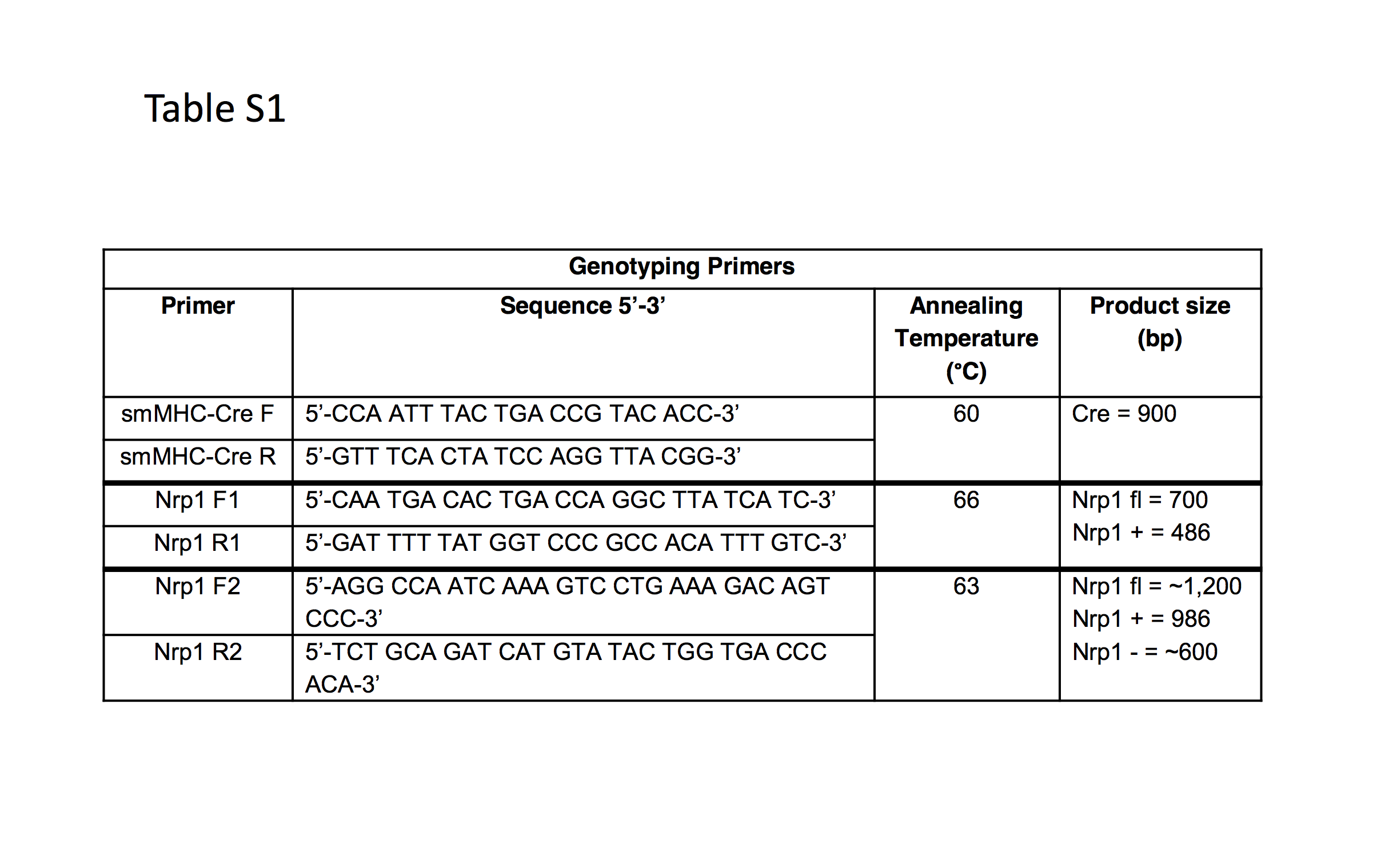

Supplement: S1 Table — (TIFF) [file pone.0115563.s010.tiff]

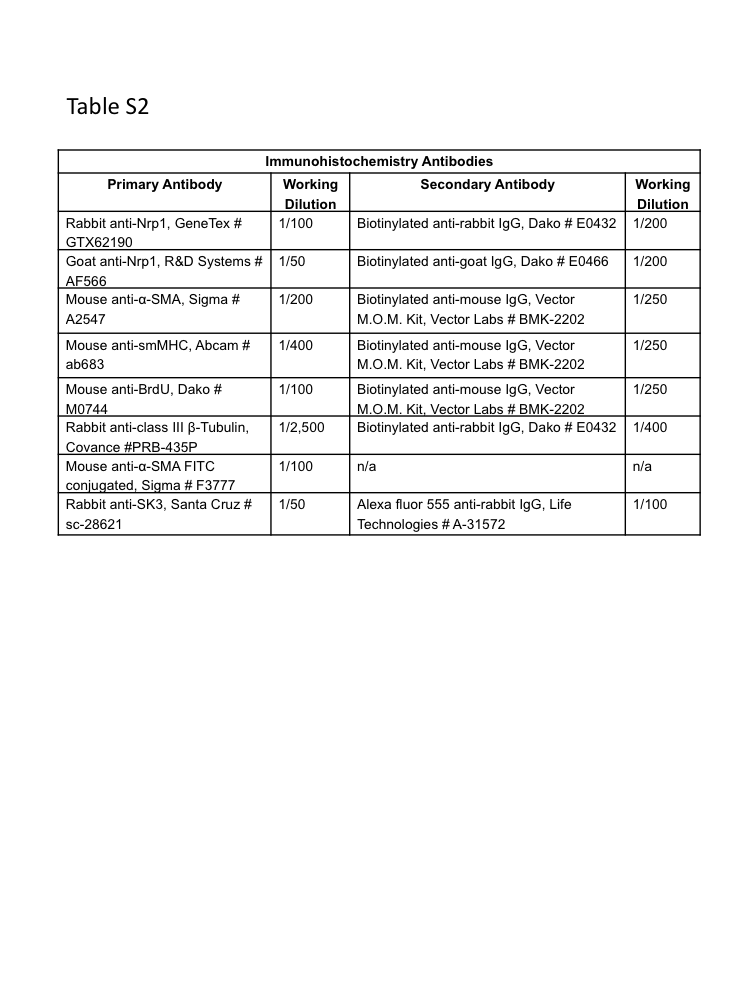

Supplement: S2 Table — (TIF) [file pone.0115563.s011.tif]

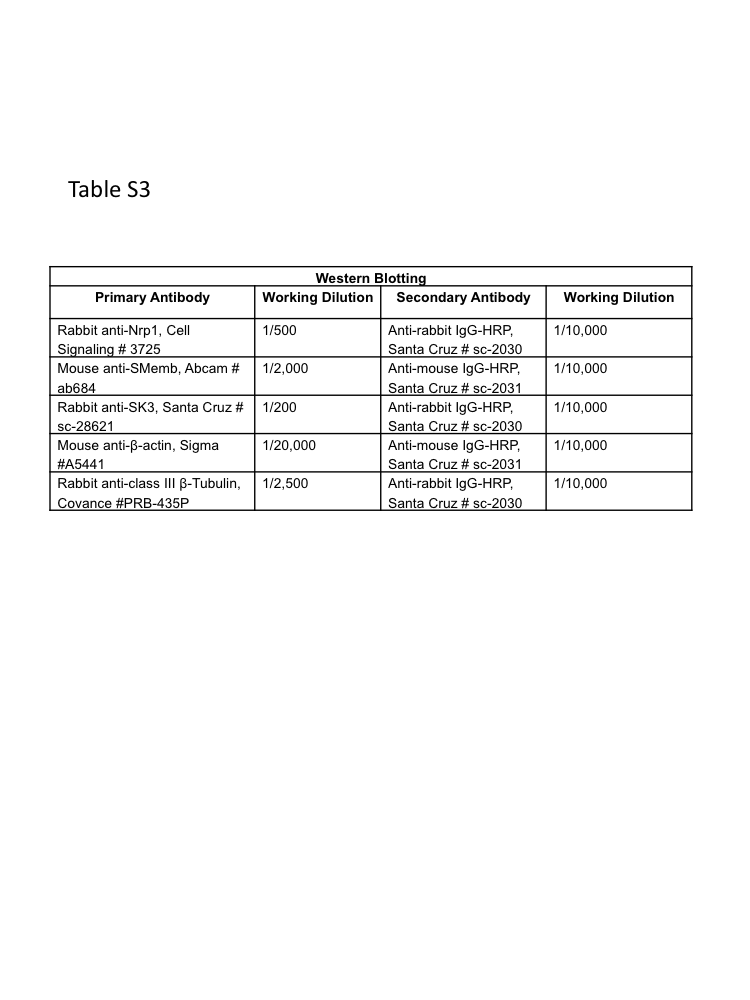

Supplement: S3 Table — (TIF) [file pone.0115563.s012.tif]

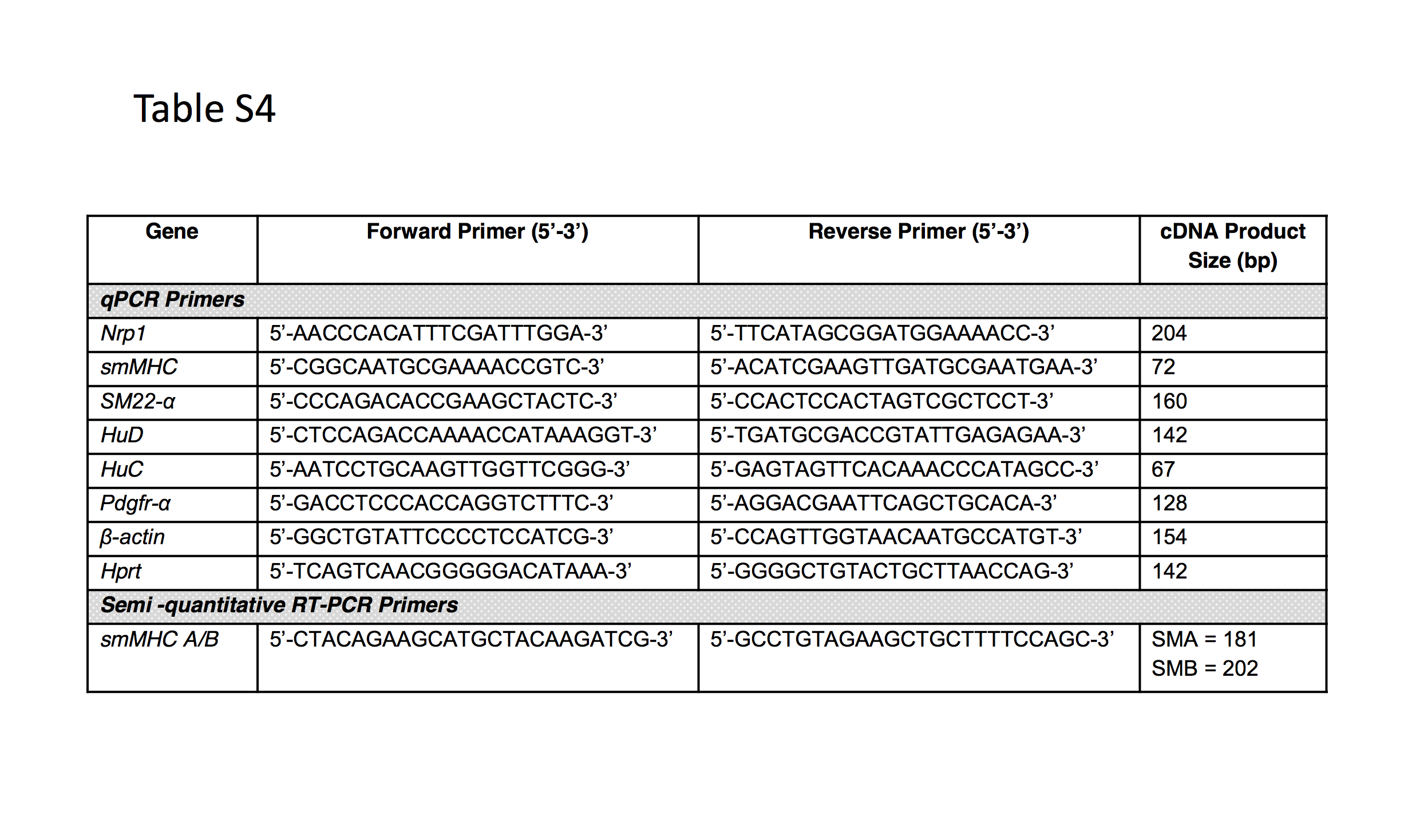

Supplement: S4 Table — All primers work at 60°C annealing temperature. (TIFF) [file pone.0115563.s013.tiff]

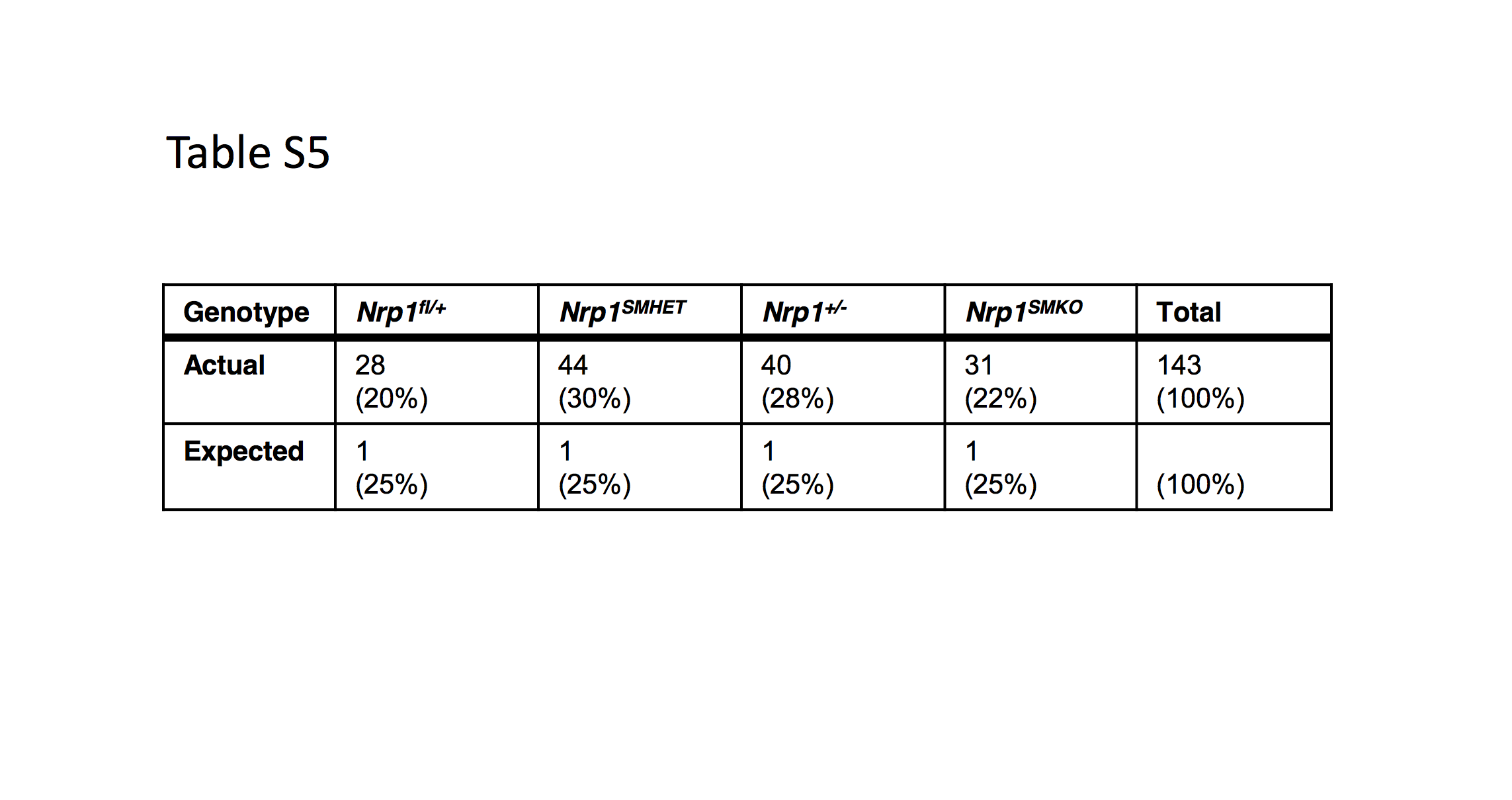

Supplement: S5 Table — Predicted and observed frequencies for each genotype, expressed as percentage of the total number of animals genotyped (total number shown in parentheses). Four different genotypes were generated: Nrp1fl/+, Nrp1SMHET, Nrp1+/− and Nrp1SMKO close to the expected Mendelian ratio of 1:1:1:1. (TIFF) [file pone.0115563.s014.tiff]
