# Supplementary material for: Neuropilin 1 Is Essential for Gastrointestinal Smooth Muscle Contractility and Motility in Aged Mice
Source: PLoS One. 2015 Feb 6;10(2):e0115563. doi: 10.1371/journal.pone.0115563 (PMC4319892; doi:10.1371/journal.pone.0115563)
Supplement: S1 File — (DOCX) [file pone.0115563.s009.docx]

**Supplemental Information**

**Materials and methods**

**Methods A: Histochemistry**

All histochemical staining was performed on paraffin-embedded tissue sections. β-galactosidase staining was performed as previously described^20^. For immunohistochemistry, organs were fixed in Histochoice^®^ Tissue Fixative (Sigma) at room temperature overnight. For chromagen-based immunohistochemistry, hydrated tissue sections (5µm) underwent endogenous peroxidase and biotin blocking, followed by blocking with 5% serum of the appropriate species. The sections were then incubated with a primary antibody overnight and with a biotinylated secondary antibody for 30 minutes. The ABC-peroxidase kit (Vector Laboratories, Inc., Burlingame, CA) was used for detection, followed by diaminobenzidine tetrahydrochloride (DAB) staining and haematoxylin counterstaining. Immunohistochemistry with a primary antibody produced in mouse was performed using M.O.M.^TM^ kits (Vector Laboratories, Inc., Burlingame, CA). For immunofluorescent staining, non-specific background staining was blocked, followed by primary and fluorescent secondary antibody incubations and mounting with ProLong® Gold antifade reagent with 4',6-diamidino-2-phenylindole (DAPI) (Life technologies Ltd., Paisley, UK). The primary antibodies and the corresponding secondary antibodies are listed in Supplementary Table S2.

**Methods B: Gene and Protein Expression**

Tissues were lysed either in Radio-Immunoprecipitation Assay (RIPA) buffer for Western blotting or in RNA*later*^®^ (Life technologies Ltd., Paisley, UK) for total RNA extraction, and homogenised using a MINILYS benchtop homogeniser (Peqlab Ltd., Sarisbury Green, UK). Protein expression in tissue homogenates was determined by Western blotting with the antibodies listed in Supplementary Table S3 using β actin as a loading control. Levels of mRNA expression were measured by qPCR using β-actin and/or HPRT as a calibrator. Experiments were performed using the Brilliant III SYBR Green qPCR kit (Agilent Technologies, # 600882) according to the manufacturer’s instructions. Gene expression data was presented using the Comparative CT method of relative quantification ^22^. For RT-PCR, PCR amplification was performed in a 20μl reaction and the amplified fragments were fractionated on a 3% Tris-Acetate-EDTA agarose gel. Primers used are listed in Supplementary Table S4.

**Methods C: Measurements of intestinal contractility**

Colonic contractility was examined in 25ml organ baths, containing aerated (95% O_2_, 5% CO_2_) Krebs-Ringer buffer (Sigma, #K0507) maintained at 37°C. Two to four colonic segments (5mm) were isolated from each animal in Krebs-Ringer buffer, mounted in the organ bath by hooks connected to force transducers and a PowerLab^®^ recording device (AD Instruments Ltd., Oxford), and allowed to equilibrate for 1 hour with three intermittent washes. During the equilibration, the colonic rings were stretched gradually to .5-1.0g tension. The segments were challenged with 80mM KCl three times with washes in between. Increasing doses of CCh or KCl were then administered to obtain dose-response curves. Changes in isometric tension were measured by the isometric transducers and recorded by LabChart^®^ software (AD Instruments Ltd., Oxford).

**Methods D: Morphometric analyses**

The area of the muscularis externa (circular and longitudinal muscle layers) was determined in cross-sections of the colon by α-SMA immunohistochemistry. The muscularis mucosae that is also detected by α-SMA staining was distinguished from the muscularis externa by structurally defining the region of the submucosa, a space filled with loose connective tissue that separates the two muscle layers.

All the bright-field images were taken using digitalised whole slide imaging scanner, Nanozoomer (Hamamatsu photonics, Japan) to capture the entire area of the colon. For most of the quantification/measurements, six randomly selected areas over the tissue of interest were selected and analysed using NDP.view2 software (Hamamatsu photonics, Japan). For BrdU-positive nuclei quantification, BrdU positive cells within the circular and longitudinal smooth muscle layers were counted from ≥3 colonic tissue cross sections per animal/tissue block.

**Methods E: Isolation of smooth muscle layers from the thoracic aorta**

The thoracic aorta was carefully dissected and collected in Hanks balanced salt solution (HBSS(1), Sigma H9269) and transferred to a digestion cocktail (collagenase type 2 (348U/ml, Worthington 4174) and elastase type 4 (3U/ml, Sigma E0258) in HBSS(2) (Gibco 14170-112)) for 5 minutes at 37°C. It was then transferred to a collecting medium (20% Fetal bovine serum/HBSS(1)) and the smooth muscle layer was carefully peeled off with forceps.
